# An *O*-Methyltransferase Is Required for Infection of Tick Cells by *Anaplasma phagocytophilum*

**DOI:** 10.1371/journal.ppat.1005248

**Published:** 2015-11-06

**Authors:** Adela S. Oliva Chávez, James W. Fairman, Roderick F. Felsheim, Curtis M. Nelson, Michael J. Herron, LeeAnn Higgins, Nicole Y. Burkhardt, Jonathan D. Oliver, Todd W. Markowski, Timothy J. Kurtti, Thomas E. Edwards, Ulrike G. Munderloh

**Affiliations:** 1 Department of Entomology, University of Minnesota, Saint Paul, Minnesota, United States of America; 2 Emerald Bio, Bainbridge Island, Washington, United States of America; 3 Seattle Structural Genomics Center for Infectious Disease, Seattle, Washington, United States of America; 4 Department of Biochemistry, Molecular Biology and Biophysics, University of Minnesota, Minneapolis, Minnesota, United States of America; Vanderbilt University, UNITED STATES

## Abstract

*Anaplasma phagocytophilum*, the causative agent of Human Granulocytic Anaplasmosis (HGA), is an obligately intracellular α-proteobacterium that is transmitted by *Ixodes* spp ticks. However, the pathogen is not transovarially transmitted between tick generations and therefore needs to survive in both a mammalian host and the arthropod vector to complete its life cycle. To adapt to different environments, pathogens rely on differential gene expression as well as the modification of proteins and other molecules. Random transposon mutagenesis of *A*. *phagocytophilum* resulted in an insertion within the coding region of an *o*-methyltransferase (*omt*) family 3 gene. In wild-type bacteria, expression of *omt* was up-regulated during binding to tick cells (ISE6) at 2 hr post-inoculation, but nearly absent by 4 hr p.i. Gene disruption reduced bacterial binding to ISE6 cells, and the mutant bacteria that were able to enter the cells were arrested in their replication and development. Analyses of the proteomes of wild-type versus mutant bacteria during binding to ISE6 cells identified Major Surface Protein 4 (Msp4), but also hypothetical protein APH_0406, as the most differentially methylated. Importantly, two glutamic acid residues (the targets of the OMT) were methyl-modified in wild-type Msp4, whereas a single asparagine (not a target of the OMT) was methylated in APH_0406. *In vitro* methylation assays demonstrated that recombinant OMT specifically methylated Msp4. Towards a greater understanding of the overall structure and catalytic activity of the OMT, we solved the *apo* (PDB_ID:4OA8), the S-adenosine homocystein-bound (PDB_ID:4OA5), the SAH-Mn^2+^ bound (PDB_ID:4PCA), and SAM- Mn^2+^ bound (PDB_ID:4PCL) X-ray crystal structures of the enzyme. Here, we characterized a mutation in *A*. *phagocytophilum* that affected the ability of the bacteria to productively infect cells from its natural vector. Nevertheless, due to the lack of complementation, we cannot rule out secondary mutations.

## Author Summary

Since its discovery in 1994, Human Granulocytic Anaplasmosis (HGA) has become the second most commonly diagnosed tick-borne disease in the US, and it is gaining importance in several countries in Europe. HGA is caused by *Anaplasma phagocytophilum*, a bacterium transmitted by black-legged ticks and their relatives. Whereas several of the molecules and processes leading to infection of human cells have been identified, little is known about their counterparts in the tick. We analyzed the effects of a mutation in a gene encoding an *o-*methyltransferase that is involved in methylation of an outer membrane protein. The mutation of the OMT appears to be important for the ability of *A*. *phagocytophilum* to adhere to, invade, and replicate in tick cells. Several tests including binding assays, microscopic analysis of the infection cycle within tick cells, gene expression assays, and biochemical assays using recombinant OMT strongly suggested that the mutation of the *o-*methyltransferase gene arrested the growth and development of this bacterium within tick cells. Proteomic analyses identified several possible OMT substrates, and in vitro methylation assays using recombinant *o-*methyltransferase identified an outer membrane protein, Msp4, as a specifically methyl-modified target. Our results indicated that methylation was important for infection of tick cells by *A*. *phagocytophilum*, and suggested possible strategies to block transmission of this emerging pathogen. The solved crystal structure of the *o-*methyltransferase will further stimulate the search for small molecule inhibitors that could break the tick transmission cycle of *A*. *phagocytophilum* in nature.

## Introduction

*Anaplasma phagocytophilum* is an obligately intracellular bacterium classified in the order *Rickettsiales*, and is the causative agent of Human Granulocytic Anaplasmosis (HGA) [1]. HGA is characterized by high fevers, rigors, generalized myalgias, and severe headache. It is a potentially life-threatening disease, with 36% of patients diagnosed with HGA requiring hospitalization, 7% needing urgent care, and mortality of ~1% [2]. The incidence of HGA has been increasing steadily, from 348 identified cases in 2000 when it first became reportable to the CDC, to 1761 cases in 2010 [3] and 2,782 reported cases in 2013 [4]. Similar trends are evident in other countries in Europe and Asia [reviewed in [5]]. In addition, *A*. *phagocytophilum* infects domestic animals such as dogs, cats, and horses, as well as wild mammals from deer and wolves to various rodents [6].

*A*. *phagocytophilum* is transmitted by ticks of the *Ixodes ricinus* complex, with *Ixodes scapularis* and *Ixodes pacificus* being the most important vectors in the USA [7]. Transovarial transmission does not occur in these ticks, and has only been reported in tick species and *A*. *phagocytophilum* strains that are not implicated in human disease [8]. The natural transmission cycle involves acquisition of the pathogen from small wild rodents by tick larvae, and transstadial transmission to nymphs and adults that may infect a new mammalian host during a subsequent bloodmeal. Therefore, the ability of *A*. *phagocytophilum* to cycle between ticks and mammalian hosts is imperative for bacterial survival in nature [8]. The development of *A*. *phagocytophilum* in *Ixodes* sp. vector ticks remains unknown but has been described in tick cell culture where it is biphasic [9]. The time required for *A*. *phagocytophilum* to complete development in ISE6 cells differs from that observed in HL-60 and endothelial cells [9]. Adhesion to ISE6 cells started at 30 min p.i., and by 1 hr p.i the bacteria were attached to the tick cell membrane, initiating the process of endocytosis, which was probably driven by receptor-mediated interactions. By comparison, in HL-60 cell culture, >70% of bacteria were observed binding to host cells in the first 40 min p.i., and at that time, 26% of the bacteria had been internalized [10]. Internalization in tick cells began at 2 hr p.i. and was complete by 4 hr p.i., whereas in HL-60 cell culture, only 45.5% of the bacteria had entered the cells at this time point [10]. Replication by binary fission started by 8 hr p.i. in tick cells [9] whereas in HL-60 cells only a few bacteria had turned into the reticulate form by 12 hr p.i. and initiated replication [10]. Nevertheless, many of the molecular events involved in the infection of mammalian cells are known [11], but much less is known in the tick counterpart [12]. Studies to understand vector-pathogen interactions have focused on tick responses and tick factors important for successful establishment of the pathogen in ticks [13–15], but *A*. *phagocytophilum* genes and proteins that are important for development in tick cells and ticks remain largely unidentified. Some studies have examined gene expression of *A*. *phagocytophilum* during infection of *I*. *scapularis* ticks or *I*. *scapularis* ISE6 cells, but have focused on certain periods, such as transmission feeding or late phases of replication [16,17]. As a result, little is known about the proteins, and their modifications, necessary for the early phases of infection of tick vector cells by *A*. *phagocytophilum*. Survival in dissimilar hosts such as the arthropod vector and the mammal that present important biological differences requires rapid adaptation of bacteria, and involves proteins and other molecules that are differentially expressed or produced in response to host-specific cues [17]. Thus, the identification of such factors is crucial to our understanding of the biology of this important pathogen.

Analyses of *A*. *phagocytophilum* gene expression and proteomics [16] may fail to identify proteins that are not abundant or not directly involved in infection but still play an important role. The intracellular nature of *A*. *phagocytophilum* has made it difficult to study the function of genes involved in intracellular invasion and replication using genetic techniques such as homologous recombination. Nevertheless, random mutagenesis of *A*. *phagocytophilum* using the *Himar1* transposase system [18] has become an important tool to probe gene function in these and related bacteria [19–21]. Here, we analyzed a mutant, referred to as ΔOMT, with a transposition into genomic locus APH_0584 that contains a gene encoding a member of family 3 S-adenosyl methionine (AdoMet or SAM)-dependent *o*-methyltransferases. Transcription of genomic locus APH_0584 was barely detected in HL-60, HMEC-1 or ISE6 cells during late phases of infection [17]. However, the mutation of this gene rendered the bacteria unable to efficiently colonize *I*. *scapularis* (ISE6) cells.

Methyltransferases are involved in important bacterial activities such as cell signaling, cell invasion, and gene expression, as well as in metabolic pathways and pathogenesis [22–24]. They participate in the modification of membrane components, cofactors, signaling and defense compounds [23], and have been linked to virulence in several bacteria [25–27], fungi [28], and viruses [29]. OmpB proteins from several rickettsial pathogens are methylated at multiple residues by lysine methyltransferases [30], although recombinant OmpB produced by *E*. *coli* in the absence of a lysine methyltransferase has been shown to mediate adhesion and invasion of HeLa cells in a Ku70-dependent manner [31]. Methylation of glutamic acid residues in the outer membrane protein OmpL32 of *Leptospira interrogans* is thought to be involved in its virulence and ability to colonize liver and kidney cells in hamsters [32]. To gain insights into its overall structure and the interaction of the *A*. *phagocytophilum o*-methyltransferase with cofactors, we solved the crystal structure of the *apo*-enzyme, the enzyme bound to S-adenosine homocysteine (SAH), to SAH and manganese, and to SAM and manganese. This revealed large differences with the nearest homolog in the PDB (*o*-methyltransferase from the cyanobacterium *Synechocystis* sp.; PDB ID: 3CBG). Here, we analyzed the phenotypic and proteomic changes that characterized ΔOMT, and present evidence that the *o*-methyltransferase is involved in adherence to and necessary for replication of *A*. *phagocytophilum* in tick cells.

## Results

### Disruption of aph_0584 encoding an OMT results in phenotypic changes

**Fig 1:**
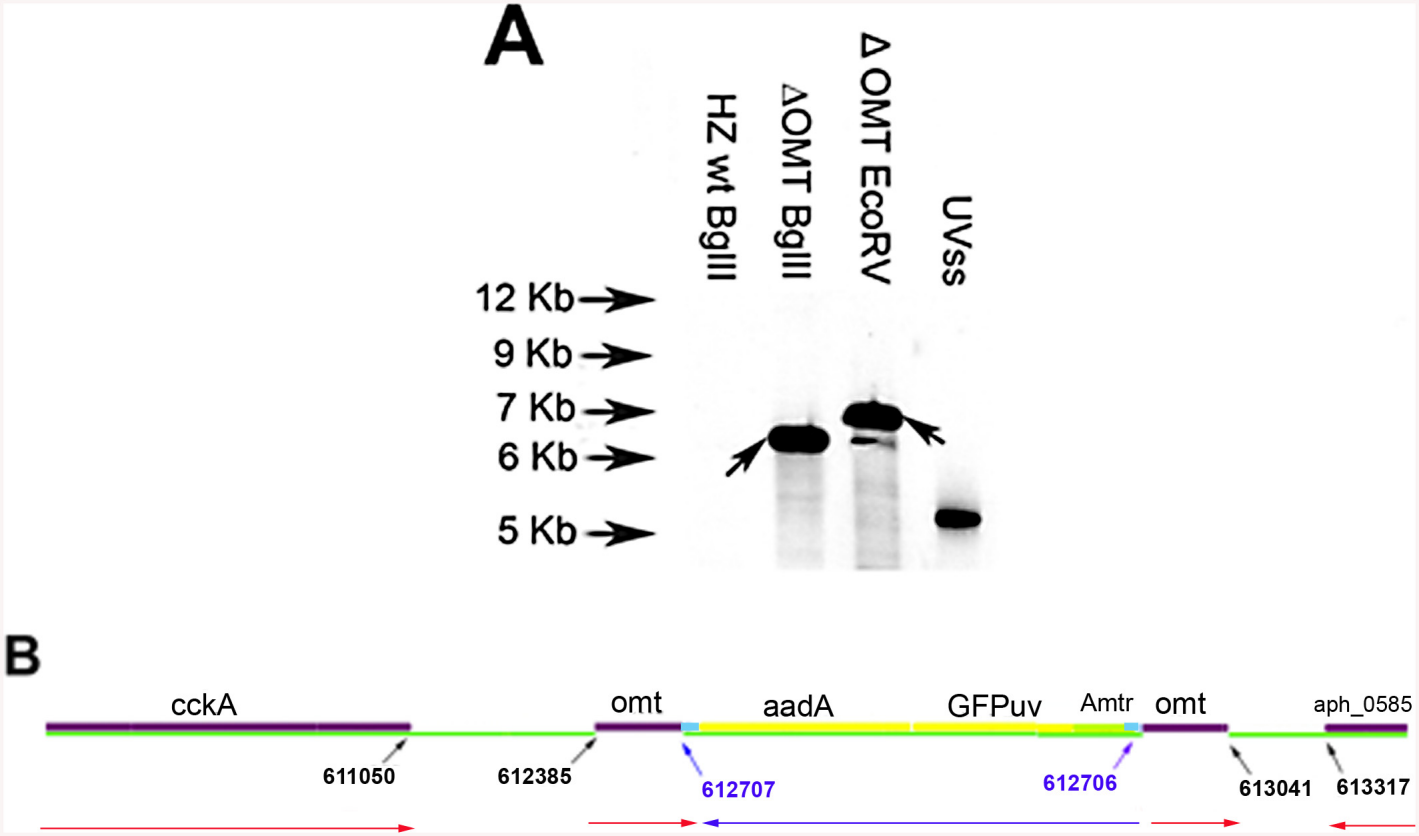
Determination of transposon insertion site in ΔOMT. A) Southern blot of ΔOMT DNA hybridized with a digoxigenin-labeled probe specific for *gfp*_uv_ encoded by the transposon (black arrows) to determine the number of insertion sites in the population. From left to right, lanes contain DNA from the following samples: First lane DNA from wild type bacteria digested with *Bgl*II, (negative control); second lane, ΔOMT DNA digested with *Bgl*II; third lane, ΔOMT DNA digested with *EcoR*V. The second and third lanes presented a single band at around 7 Kb, indicating a single insertion event in the population. The fourth lane contains 1 pg of the undigested DNA encoding *gfp*_uv_ and *aadA* (positive control). B) Graphic representation of the insertion site of the transposon encoding the Amtr promoter (green), the *gfp*_uv_ gene (yellow), and the spectinomycin resistance gene *aadA* (yellow), within the *himar* repeats (light blue). The coding region of the *o*-methyltransferase gene *APH_0584* (purple) was interrupted at position 612707–612706 (blue arrows). Red arrows represent the direction of transcription for each gene within the *A*. *phagocytophilum* genome, and the direction of transcription for the inserted genes (blue arrow). Transposition occurred ~1600 bp upstream from the sensor kinase gene (cckA: APH_0582) and ~800 bp downstream from a hypothetical gene encoding APH_0585.

The ΔOMT, selected and maintained in HL-60 cells, was unable to grow in ISE6 cells. The mutant expressed the Green Fluorescent Protein (GFPuv) from a *Himar1* transposon [18] and Southern blot analysis identified a single insertion site (Fig 1A). Digestion of ΔOMT DNA with *Bgl*II yielded a single band hybridizing to the probe, suggesting a clonal population (Fig 1A), although *EcoR*V yielded several smaller bands that were most likely due to incomplete digestion. Recovery of the transposon along with flanking sequences from ΔOMT DNA by restriction enzyme digestion and cloning indicated transposition into *aph_0584* (Gene ID: 3930223; *o*-methyltransferase 3 family member) between nucleotide positions 612707–612706 of the *A*. *phagocytophilum* strain HZ genome sequence ([33]; Fig 1B). The single insertion event suggested that the changes in phenotype were due to the disruption of that particular gene.

**Fig 2:**
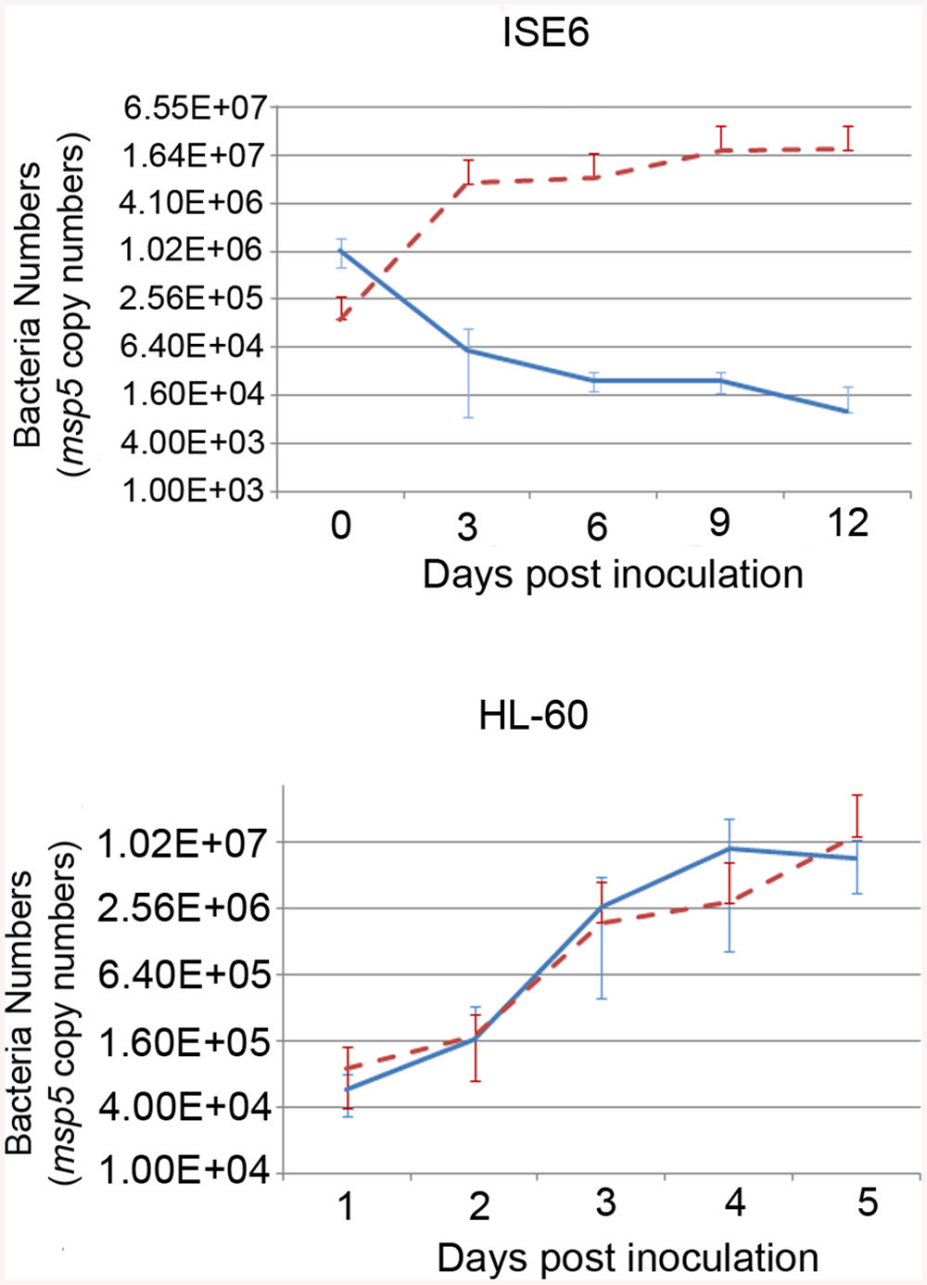
Effects of the mutation on the growth of *A*. *phagocytophilum* in tick cell culture. Growth curves representing the replication of ΔOMT (solid blue line) and wild-type (dashed red line) bacteria in ISE6 (top) or HL-60 cells (bottom). ΔOMT and wild-type bacteria were purified from HL-60 cells and inoculated into ISE6 or HL-60 cultures. The number of bacteria was estimated by determining the copy number of the *msp5* gene. Each data point represents the average number of bacteria from triplicate samples, and vertical bars indicate the standard deviation. Statistical differences were evaluated by repeated measures ANOVA. ΔOMT bacteria were not able to replicate in ISE6 cells (top) and had decreased already by day 3, which was significantly different from the replication of wild-type bacteria in ISE6 cells (P = 0.008). This is in contrast to ΔOMT growth within HL-60 cells (bottom), in which there was no significant difference between mutant and wild-type bacteria numbers (P = 0.504).

To determine the mechanism whereby the mutation affected the phenotype of *A*. *phagocytophilum*, we compared wild-type and mutant bacteria with respect to their ability to invade and replicate in tick and mammalian host cells, determined the timing of wild-type OMT expression and its localization, identified the protein methylated by the enzyme as well as cofactors, and solved the crystal structure of the OMT. First, we analyzed the growth of the mutant in HL-60 and ISE6 cells and compared it to wild-type bacteria under the same conditions. The ΔOMT was not able to replicate in ISE6 cells and qPCR showed that *msp5* (a single copy gene used as a proxy for bacterial numbers) copy numbers decreased significantly over a 12-day period (P = 0.008) (Fig 2). This was in contrast to the behavior of ΔOMT bacteria in HL-60 cells, in which they were able to multiply in a manner comparable to wild-type bacteria (Fig 2, P = 0.504). Only the datasets for the 1:16 dilution in ISE6 and 1:100 in HL-60 are shown, but other dilutions presented the same tendency.

### Omt mutation and AdOx treatment reduced *A*. *phagocytophilum* binding to ISE6 cells

**Fig 3:**
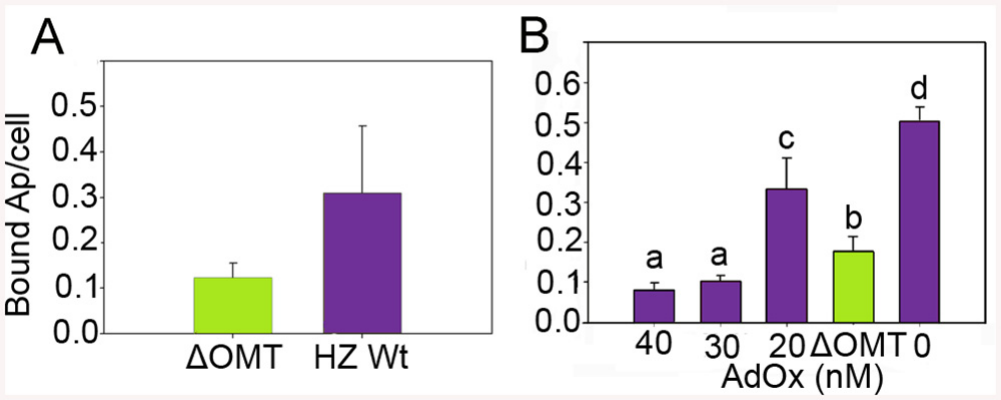
Reduced binding of *A*. *phagocytophilum* to ISE6 cells caused by *omt* mutation or inhibition with AdOx. A) Mutation of *omt* (ΔOMT; green bar) in *A*. *phagocytophilum* HZ caused a reduction in the number of bacteria adhering to tick cells when compared to the number of wild-type bacteria per tick cell (purple bar). Bacteria purified from HL-60 cells were incubated with ISE6 cells for 30 min at room temperature. Unbound bacteria were removed using vigorous washes and the remaining attached bacteria were counted using immunofluorescence microscopy. The difference in binding was statistically significant (t-value = -4.1011; P = 0.0001). Bars represent the average number of adherent bacteria per cell, and vertical lines indicate the standard error of the mean. B) Effect of AdOx (Adenosine dialdehyde; an inhibitor of SAM-depended methyltransferases) on adherence of *A*. *phagocytophilum* HZ wild-type to ISE6 cells in comparison to the effect of mutation of the *omt* gene in ΔOMT bacteria. Wild-type *A*. *phagocytophilum* were incubated with different concentrations of AdOx for 1 hr while ΔOMT was held in medium alone, and then mixed with ISE6 cells. Bars represent the average number of bacteria bound to ISE6 cells in each treatment, and bars with the same letter are not significantly different, whereas different letters indicate a significant difference (P<0.001). The standard error of the mean from four replicates is shown as vertical lines.

Because of the rapid decline of ΔOMT numbers noticeable already during the first 24 hr of incubation with ISE6 cells, we tested the ability of the mutant to bind to ISE6. There was a significant reduction (>50%) in binding of ΔOMT (t-value = -4.1011; P = 0.0001) to ISE6 cells from 0.3 bacteria per cell in the wild type to 0.12 mutant bacteria per cell (Fig 3A). To support these results, an inhibitor of SAM-dependent methyltransferases was used to reproduce the effects of the lack of methylation brought about by the disruption of *omt* on binding of *A*. *phagocytophilum* to ISE6 cells. Wild-type bacteria were pre-incubated with 20 nM, 30 nM, and 40 nM of adenosine periodate (AdOx) for 1 hr before addition to ISE6 cells and incubated for another hr; untreated bacteria served as controls. All concentrations of AdOx affected the ability of *A*. *phagocytophilum* to bind to ISE6 cells significantly (P<0.001) (Fig 3B). In controls, an average of 0.529 bacteria bound per cell, compared to ΔOMT with only 0.156 bacteria per cell. 20 nM AdOx decreased binding to 0.307 bacteria per cell, which was less than the reduction in attachment observed in ΔOMT (Fig 3B). However, as the concentration of AdOx increased to 30 nM and 40 nM, the effects were stronger than in ΔOMT with only 0.093 and 0.086 bacteria bound per cell, respectively, and these differences were statistically significant when compared to the ΔOMT and the 20 nM AdOx concentration (Fig 3B). These results corroborated the effects of the inhibition of methylation due to the mutation of *omt* on *A*. *phagocytophilum* binding. The greater inhibition of bacterial binding when using 30 nM and 40 nM of AdOx was probably due to inhibition of other methyltransferases. To test this hypothesis, we pre-incubated ΔOMT bacteria with AdOx before addition to ISE6 cells, as described for wild-type bacteria. Incubation of the ΔOMT with 20 nM and 40 nM did not significantly decrease binding to ISE6 cells (S1 Fig), suggesting that no other methyltransferases were involved in tick cell invasion.

### Omt is up-regulated during binding and internalization, and the protein localizes to bacteria interacting with tick cells

**Fig 4:**
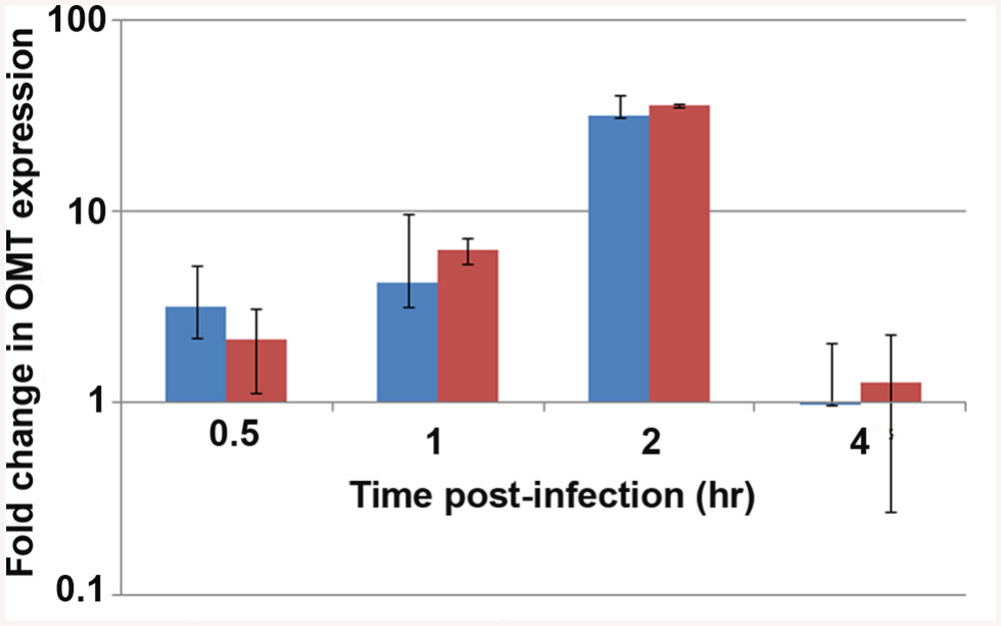
Expression of the OMT during infection of ISE6 cells. qRT_PCR to track the expression of the *omt* gene in wild-type *A*. *phagocytophilum* during adhesion and invasion of ISE6 cells in comparison to *omt* gene transcripts detected in bacteria interacting with HL-60 cells. Wild-type bacteria were purified from HL-60 cells and inoculated onto cell layers of ISE6 cells or mixed with suspended HL-60 cells. RNA was purified at the indicated times post inoculation, and qRT-PCR was performed. The bars represent the average of the fold change normalized to *msp5* (blue) or to *rpoB* (red), and vertical lines represent the standard error of the mean. Up-regulation of *omt* transcription was seen as early as 30 min, reaching 34-fold change at 2 hr. At 4 hr there was no detectable difference in bacterial gene expression between the two host cell types (0.97 fold change).

Because of the effects on binding to tick cells seen in ΔOMT, we tested the expression of the *omt* gene by qRT-PCR during early stages of wild-type *A*. *phagocytophilum* interaction with and development in ISE6 cells, using *rpoB* and *msp5* genes as normalizers. In our discussion, we focused on the fold change of *omt* normalized to *msp5*, but normalization against either gene showed the same trend (Fig 4). Up-regulation of the *omt* gene started at 30 min post-inoculation (p.i.), continued to increase from 3-fold at 30 min to 5-fold by 1 hr p.i., and by 2 hr, the gene reached its maximum expression, showing 34-fold up-regulation compared to bacteria entering HL-60 cells (Fig 4). At 4 hr, *omt* expression decreased to 0.97-fold change (Fig 4), similar to that seen in bacteria infecting HL-60 cells. Our results are congruent with electron microscopy based studies that showed *A*. *phagocytophilum* bound to tick cells between 30 min and 1 hr p.i. and cell entry at 2 hr p.i., the time when we saw maximum *omt* gene expression [9].

**Fig 5:**
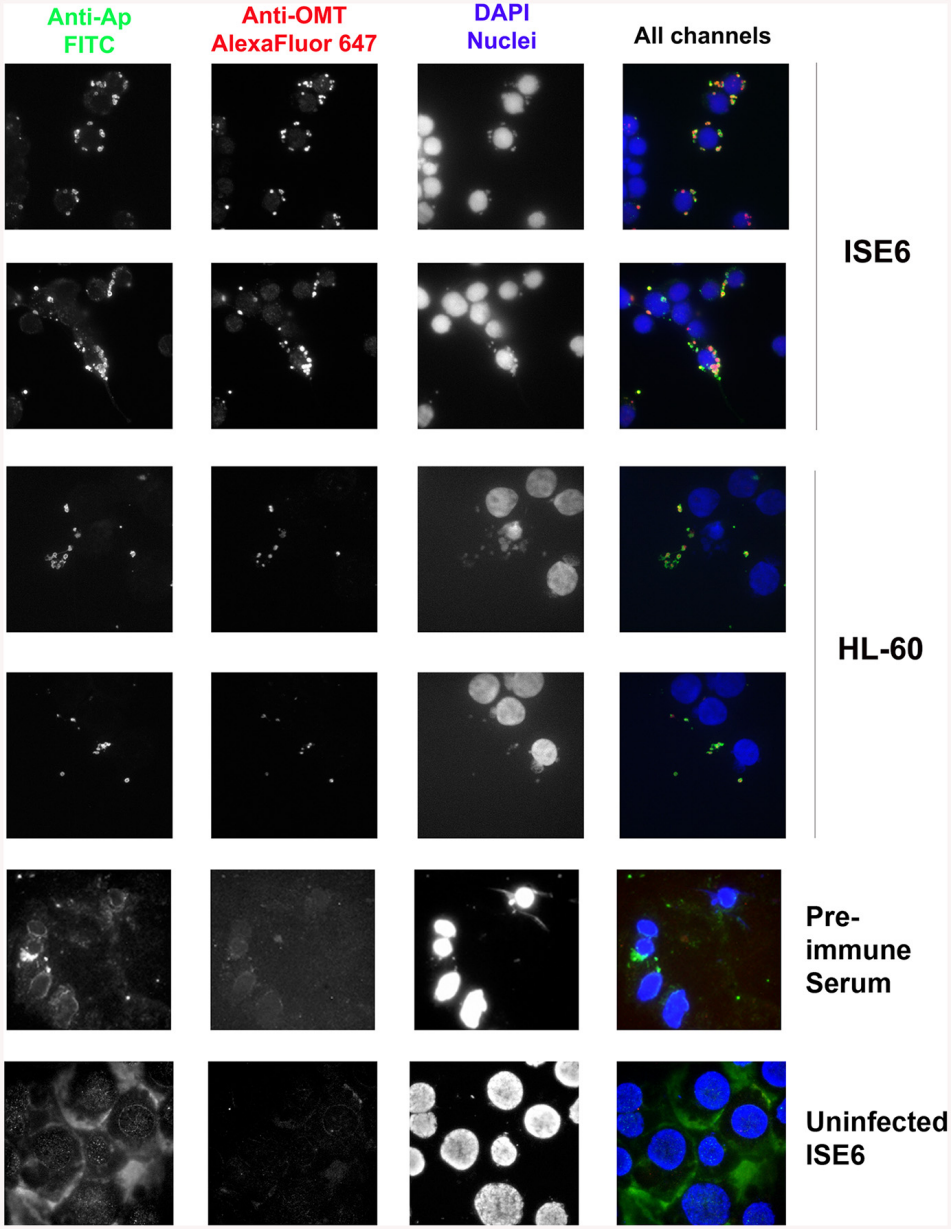
OMT is highly expressed by *A*. *phagocytophilum* binding to ISE6 cells and not HL-60 cells. Wild-type *A*. *phagocytophilum* HZ bacteria isolated from HL-60 cells were incubated with ISE6 (tick) and HL-60 (human) cells for 2 hr to identify the conditions under which OMT was detectable by immunofluorescence microscopy. Cells with bound bacteria were fixed and sequentially incubated with polyclonal dog anti-*A*. *phagocytophilum* antibodies and monospecific mouse anti-rOMT antibodies, and stained with FITC-labeled anti-dog antibodies (green channel), and anti-mouse AlexaFluor647-labeled antibodies (red channel). DAPI (blue channel) was used to label host nuclei. *A*. *phagocytophilum* HZ expressing OMT bound both labels resulting in a yellow to red signal. High expression of OMT (red signal) was observed labeling bacteria bound to ISE6 cells. No significant red signal was observed in bacteria bound to HL-60 cells. Pre-immune serum and uninfected ISE6 cells were used as controls.

Because *omt* expression correlated with binding and entry of the bacteria to ISE6 cells, and the mutation of this gene affected the ability of the bacteria to bind to these cells, we investigated the localization of the protein during this step in cell infection. Mouse antiserum against recombinant OMT (rOMT) was produced to label the protein during binding of wild-type *A*. *phagocytophilum* to ISE6 (2 hr p.i.), using an immunofluorescence assay (IFA). OMT was detected with mouse anti-rOMT serum followed by incubation with anti-mouse IgG conjugated to AlexaFluor647 (red fluorescence). All bacteria were labeled with dog anti-anaplasma serum followed by incubation with fluorescein isothiocyanate (FITC)-conjugated anti-dog IgG (green fluorescence). Bacteria interacting with ISE6 showed strong OMT expression while bacteria interacting with HL-60 showed only slight expression (Fig 5). This was in agreement with the 34-fold up-regulation of the gene seen by qRT-PCR during adhesion to ISE6 cells (Fig 4). Bacteria incubated with pre-immune serum did not fluoresce red nor did uninfected ISE6 cells incubated with anti-rOMT antibodies followed by TRITC-conjugated anti-mouse IgG, demonstrating that the serum specifically labeled OMT.

### ΔOMT bacteria are internalized but unable to replicate within tick cells

We conducted a time course comparison of wild-type versus ΔOMT bacteria to identify the stage at which the infection process failed. To increase the chances of detecting differences between the intracellular development of the ΔOMT and wild-type bacteria, we performed an optimized binding assay in which a sparse monolayer of adherent ISE6 cells growing in MatTek dishes was exposed to numerous bacteria (100–300 bacteria/cell) and washed gently to maximize retention of bacteria bound to the cells. This is in contrast with our previous assay that used suspended ISE6 cells, a lower multiplicity of infection (MOI), and vigorous washes so that only strongly bound bacteria remained, which increased the sensitivity of the assay but made it difficult to track intracellular development of ΔOMT.

**Fig 6:**
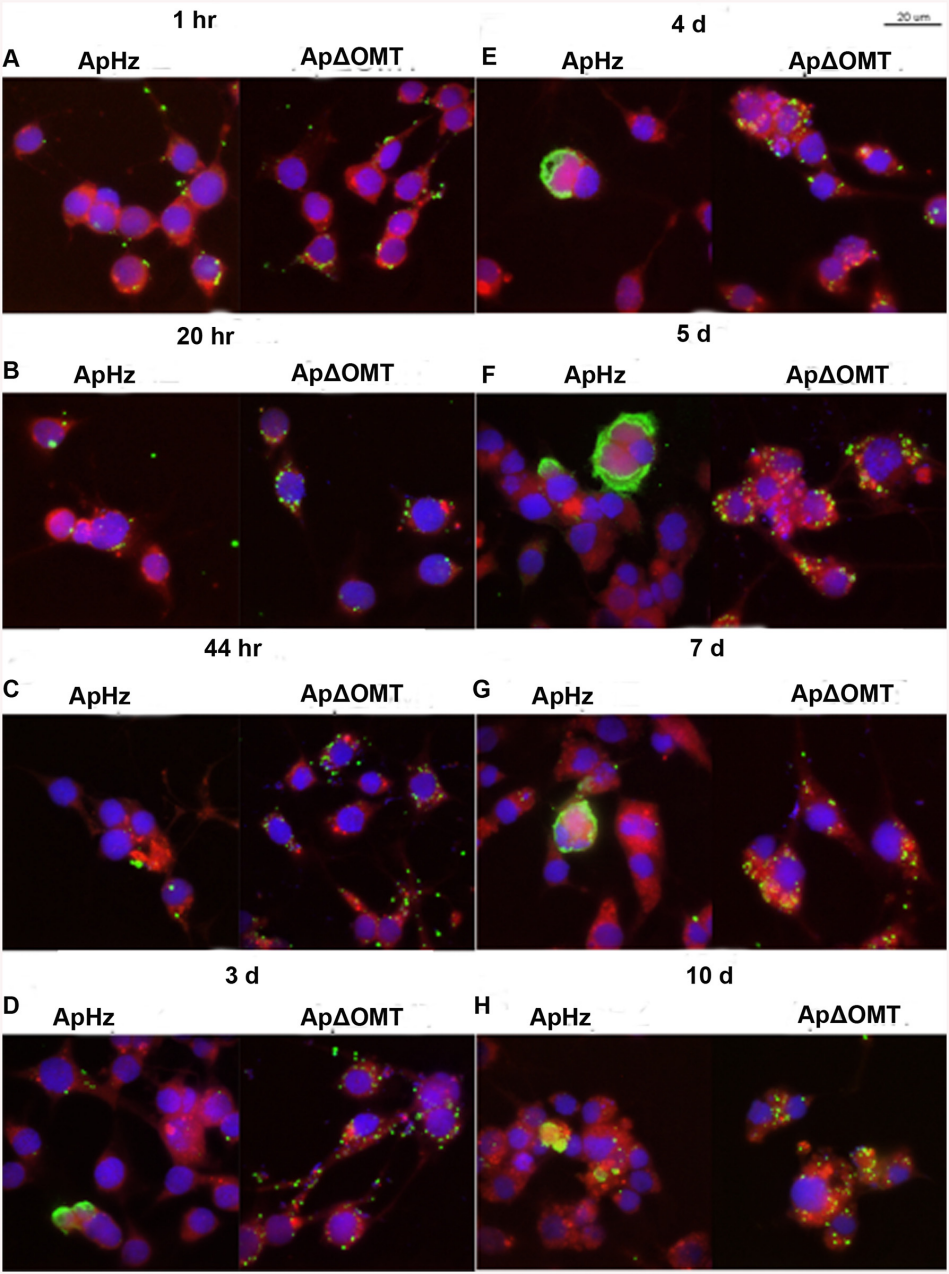
Time course of wild type and ΔOMT *A*. *phagocytophilum* development within ISE6 cells Host cell-free wild-type (ApHZ) and mutant (ApΔOMT) *A*. *phagocytophilum* were harvested from HL-60 cells and incubated with ISE6 cells expressing mCherryLifeAct (red, to provide contrast) grown in MatTek chambers for 1 hr at a high MOI (100–300 bacteria/cell) to maximal internalization that permitted observation of phenotypic defects in the ΔOMT which was not possible using a stringent invasion assay. Subsequently, unbound bacteria were washed away and samples were taken at the indicated times post infection. Panels for each sampling time show wild-type bacteria at left, and mutant at right. A) 1 hr, B) 20 hr, C) 44 hr, D) 3 days, E) 4 days, F) 5 days, G) 7 days, and H) 10 days. Bacteria were labeled with FITC (green), cell nuclei were labeled with DAPI (blue), and samples were viewed by confocal microscopy to compare development of bacteria within ISE6 cells. Saturated binding allowed sufficient numbers of ApΔOMT to be internalized (1 hr panels) so that their development could be visually tracked for 10 days. From 44 hr through 10 days, small to very large morulae developed in ApHZ, while the ΔOMT persisted as individual bacteria within the ISE6 cells and never formed morulae. The size bar represents 20 μm.

The development of the ΔOMT was compared to wild-type bacteria using confocal microscopy of fixed and immunofluorescently labeled ISE6 cells in MatTek dishes after 1 hr of exposure to bacteria, and subsequently on days 1, 2, 3, 4, 5, 7, and 10. With this method there were no differences observed in binding or internalization between the ΔOMT and wild-type bacteria (Fig 6A and 6B). However, by 44 hr, wild-type bacteria started to form morulae, whereas ΔOMT bacteria remained singly within the cells (Fig 6C). By days 3 and 4, the wild-type bacteria had formed large morulae (Fig 6D and 6E) and on days 5–10, wild-type infections had become asynchronous, with bacteria from lysed cells infecting new cells while other cells harbored large morulae (Fig 6F–6H). ΔOMT bacteria, however, never developed morulae during the 10 days of observation. Only single bacteria were observed within the infected cells throughout (Fig 6D–6H), suggesting that the mutant bacteria were unable to replicate and form morulae within the infected cells even though they were successfully internalized. Observation of ΔOMT bacteria by confocal microscopy identified their location as intracellular.

To confirm that in fact the ΔOMT bacteria resided inside the tick cells, we performed a trypsin-protection assay, similar to those used to remove uninternalized bacteria and/or beads from cells to examine binding proteins in *Ehrlichia chaffeensis*, *A*. *phagocytophilum*, and *Helicobacter pylori* [34–36]. Cultures were used four days after inoculation with either mutant or wild-type bacteria, when wild-type bacteria had formed large intracellular morulae (S2A–S2C, S2B and S2C Fig), whereas mutants persisted as individual intracellular bacteria (S2D, S2E and S2F Fig). Cultures were treated with trypsin once (wild-type and mutant) or twice (mutant only) to remove any extracellular bacteria, and untrypsinized cells scraped off the growth substrate were used for comparison. As expected, neither mechanical scraping (S2A and S2B Fig) nor trypsinization affected the wild-type morulae (arrow heads) already formed within ISE6 cells (S2C Fig). Similarly, when ISE6 cultures that had been exposed to ΔOMT bacteria for four days were scraped off the flask (S2D Fig), trypsinized once (S2E Fig) or twice (S2F Fig), there was no effect on the bacteria, confirming that they were located intracellularly as also indicated by confocal microscopy. As observed in the previous experiment, mutant bacteria remained as individuals (arrows) within the infected cells and were unable to develop to morulae. Additional controls demonstrated that wild-type bacteria adherent to ISE6 cells for 1 hr were removed from the cells by trypsinization (S2G and S2H Fig), another indication that single ΔOMT bacteria visualized four days p.i. had been internalized. Note that host cell nuclei (asterisks) were recognized by the dog anti-*A*. *phagocytophilum* serum and subsequently labeled by the secondary FITC-conjugated anti-dog antibody (S2D, S2E and S2F Fig). They were not detected in panels A, B, and C because the brightness of the large wild-type morulae required a shorter exposure during image acquisition than that used to image ΔOMT. Anti-nuclear antibodies have been detected in dogs infected with vector-borne pathogens (Smith et al. 2004), explaining the reactivity of the dog’s antiserum with host cell nuclei.

### Mutation of omt affects abundance of several *A*. *phagocytophilum* proteins

To globally identify proteins that were differentially represented in the ΔOMT compared with the wild-type bacteria, we used a proteomic approach based on iTRAQ (isobaric tag for relative and absolute quantitation) technology. Peptides in each sample were labeled with different isotopic tags of known mass to quantify the relative abundance of the proteins in each sample. Both ΔOMT and wild-type bacteria were incubated with ISE6 cells for 4 hr at 34°C. Proteins were extracted from bacteria released from host cells, and triplicate samples were analyzed by tandem mass spectrometry (MS/MS) [37]. In each replicate, multiple *A*. *phagocytophilum* proteins were identified that appeared to be differentially abundant in the mutant (S1 Text). Of these, 23 *A*. *phagocytophilum* proteins (Table 1) were identified as differentially abundant in all replicates: five proteins were less abundant (hypothetical protein APH_0406, major surface protein 4, anti-oxidant AhpCTSA family protein, and ankyrin (GI88607707)), and 19 appeared more abundant (Table 1). Hypothetical protein APH_0406, and major surface protein 4 (Msp4) presented the lowest relative expression ratios (both <0.2), indicating that they were highly expressed in wild-type bacteria during binding to ISE6 cells compared to the mutant (Table 1). Several proteins known to be involved in infection of mammalian cells [38,39], or highly expressed in *A*. *phagocytophilum* replicating in human cells [17], were more abundant in the mutant (Table 1). These proteins included several membrane proteins (P44-18ES, an OmpA family protein, P44-1 Outer membrane protein, an OMP85 family outer membrane protein, and hypothetical protein APH_0405) as well as stress response proteins (co-chaperone GrpE, chaperonin GroEL, and chaperone DnaK) (Table 1). This suggested that, unlike wild-type *A*. *phagocytophilum*, the ΔOMT failed to respond to interaction with ISE6 cells in a host cell specific manner, and as a result, the repertoire of proteins in its outer membrane remained unchanged. It is also possible that lack of OMT activity disrupted an environmentally responsive regulatory mechanism or sensor that prepares *A*. *phagocytophilum* for changes in hosts. Correct quantification of proteins by iTRAQ is problematic (Shirran and Blotting 2010), and to confirm these results, we examined transcription of several genes that were more abundant in ΔOMT than wild-type during bacterial adhesion to tick cells (based on the iTRAQ data) using qRT-PCR. RNA was isolated from ΔOMT and wild-type bacteria purified from HL-60 cells during late stages of infection to investigate transcription before exposure to ISE6 cells, and after a 2 hr incubation with ISE6 cells for comparison. In HL-60 cells, genes encoding OmpA, p44-18ES, and APH_0404 were strongly up-regulated 19-, 267-, and 5-fold, respectively compared to values obtained after 2 hr in ISE6 cells (S3 Fig). Genes encoding APH_0405 and cytochrome C oxidase subunit II were not regulated (1.5 and 1.3 fold average difference, respectively (S3 Fig)), whereas *msp4* expression was down-regulated (0.0033 fold average down-regulation in both the ΔOMT and wild-type bacteria) compared to wild-type bacteria in ISE6 cells (S3 Fig). Thus, the transcript levels mirrored the protein expression detected using iTRAQ, and suggested that the ΔOMT was not able to change gene expression to adapt to conditions in ISE6. In HL-60, the ΔOMT and wild-type bacteria had similar transcript levels, indicating that the mutation did not affect the expression of these genes.

**Table 1:** *A*. *phagocytophilum* HZ proteins that are differentially abundant in the ΔOMT, according to iTRAQ results.

Accession Number	Protein ID	Function	Average Peptides*	Ratio Protein Average ΔOMT /Wt**
GI88607117	Hypothetical protein APH_0406	Hypothetical porin	16	0.1773
GI88607879	Major Surface Protein 4	Hypothetical porin	11	0.1190
GI88607183	Anti-oxidant AhpCTSA family protein	Signal transduction	6	0.8069
GI88607707	Ankyrin	Host interactions	7	0.5898
GI88606723	Chaperonin GroEL	Stress response Moonlighting	44	1.5489
GI88607549	Chaperone DnaK	Stress response Moonlighting	31	1.1503
GI88607105	DNA-directed RNA polymerase beta subunit	Transcription	16	1.3672
GI88607442	Bifunctional proline dehydrogenase/pyrroline-5-carboxylate dehydrogenase	Amino acid metabolism	19	1.2042
GI88606872	DNA-directed RNA polymerase beta subunit	Transcription	15	1.3873
GI88607267	Hypothetical protein APH_0404	Unknown	20	1.3778
GI88607778	Polynucleotide phosphorylase/polyadenylase	RNA metabolism	15	1.5950
GI88607654	Hypothetical protein APH_0405	Membrane	24	1.3533
GI88607567	OMP85 family outer membrane protein	Membrane	11	1.2934
GI88607014	Leucyl Aminopeptidase	Protein metabolism	10	1.3933
GI88606911	Hypothetical protein APH_0906	Unknown	9	1.2636
GI88607774	F0F1 ATP synthase subunit beta	Energy metabolism Membrane proton-channel	12	1.2689
GI88607426	P44-1 Outer membrane protein	Hypothetical porin	26	1.1646
GI88607319	Translation initiation factor IF-2	Protein metabolism	3	1.2237
GI88606885	Hypothetical protein APH_1235	Unknown	3	1.3020
GI88607566	Co-chaperone GrpE	Stress response Moonlighting	4	1.3726
GI88607299	OmpA family protein	Membrane	3	1.3939
GI88607721	Cytochrome C oxidase, subunit II	Energy	3	1.6799
GI88607259	P44-18ES, expression locus with P44-18	Hypothetical porin	34	3.1262

*Average of peptides used for the quantification of the proteins.

**Ratios <1.0 are less abundant in ΔOMT and ratios >1.0 are more abundant in ΔOMT.

An analysis of the pathways affected in the ΔOMT during incubation in ISE6 based on iTRAQ data showed that several of the more abundant proteins were involved in transcription and protein metabolism, indicating that the mutant was metabolically active (Table 2). In our analysis, we only included proteins with a known role in specific pathways, according to information available at the KEGG (http://www.genome.jp/kegg/) pathways website. Hypothetical and porin proteins were not analyzed within specific pathways, since their roles have not been established.

**Table 2:** Pathways that are altered in ΔOMT during binding to and internalization into ISE6 cells.

Pathway	Pathway ID	# proteins up-regulated
RNA degradation	Aph03018	3
Pyrimidine metabolism	Aph00240	3
Purine metabolism	Aph00230	2
Nitrogen metabolism	Aph00910	2
Oxidative phosphorylation	Aph00190	2
Alanine, aspartate, and glutamate metabolism	Aph00250	2
Arginine and proline metabolism	Aph00330	2
Glyoxylate and dicarboxylate metabolism	Aph00630	1
Two-component system	Aph02020	1
Glutathione metabolism	Aph00480	1

### Identification of possible OMT substrates by iTRAQ

Because we thought it possible that the OMT might modify either bacterial or host cell proteins, *Anaplasma* and host cell peptides identified by iTRAQ as having a methyl modification were analyzed to identify those that were less abundant in ΔOMT bacteria, and in whole ΔOMT inoculated cell cultures compared to control wild-type samples. Among peptides with a <0.7 ratio of abundance between the wild-type and mutant (S2, S3 and S4 Texts), we identified eight *A*. *phagocytophilum* proteins with reduced methylation of eight corresponding peptides (Table 3). Two of the proteins, Msp4 and APH_0406, were less abundant in the mutant, by a ratio of 0.239 for Msp4 and 0.7484 for Aph_0406, and lacked methyl-modifications of specific residues. The affected amino acids were glutamic acid residues (E) in the Msp4 peptide VEVEVGYK (S2 Text), and an asparagine residue (N) in the APH_0406 peptide NVVLGGMLK (S2 Text).

**Table 3:** *A*. *phagocytophilum* proteins with reduced peptide methylation in the ΔOMT mutant identified by iTRAQ.

Accession Number	Protein ID	Peptides with modifications	Occurrence (Methylated/non-methylated)*	Peptide Ratio ΔOMT:Wt**
**GI88607441**	Branched-chain alpha-keto acid dehydrogenase subunit E2	TLSELSK Methyl(S)@6	3/2	0.6651
**GI88607727**	GTP-binding protein TypA	INSQVK Methyl (N)@2	2/4	0.5106
**GI88607117**	Hypothetical protein APH_0406	NVVLGGMLK Methyl(N)@1	5/3	0.1773
**GI88607879**	Major Surface Protein 4	VEVEVGYK Methyl(E)@4	6/11	0.109
		VEVEVGYK Methyl(E)@2	4/11	0.1178
**GI88607043**	P44-16B Outer membrane protein	TKDTAIANFSME Methyl(S)@6	3/4	0.4831
**GI88607849**	Peprotein translocase subunit SecA	RIDNQLR Methyl (D)@3	3/3	0.4966
**GI88607473**	Phosphoribosylamine-glycine ligase	VLVIGSGGR Methyl(I)@4	4/2	0.6483
**GI88607510**	RNA polymerase sigma factor RpoD	AVLADLR Methyl(D)@5	3/3	0.6772

* Number of occurrences of each methylated and non-methylated peptide.

* *Ratios <1.0 are less abundant in ΔOMT and ratios >1.0 are more abundant in ΔOMT.

Fifteen tick host cell proteins displayed reduced methylation when inoculated with the ΔOMT as compared to wild-type infected cells, but prolyl 4-hydroxylase alpha subunit (GI:240974259) and flavonol reductase/cinnamoyl-CoA reductase (GI:241703753) were the only two *I*. *scapularis* proteins to be both down regulated as well as to present peptides with reduced methylation in all replicates (S3 Table). Since no OMT was detected in ISE6 cells by IFA during infection with wild-type bacteria (Fig 5), these changes are unlikely to be due to a direct effect of the mutation, but probably reflect an absence of replicating *A*. *phagocytophilum*.

### *In vitro* assay confirms the methylation of Major Surface Protein 4 (Msp4)

**Fig 7:**
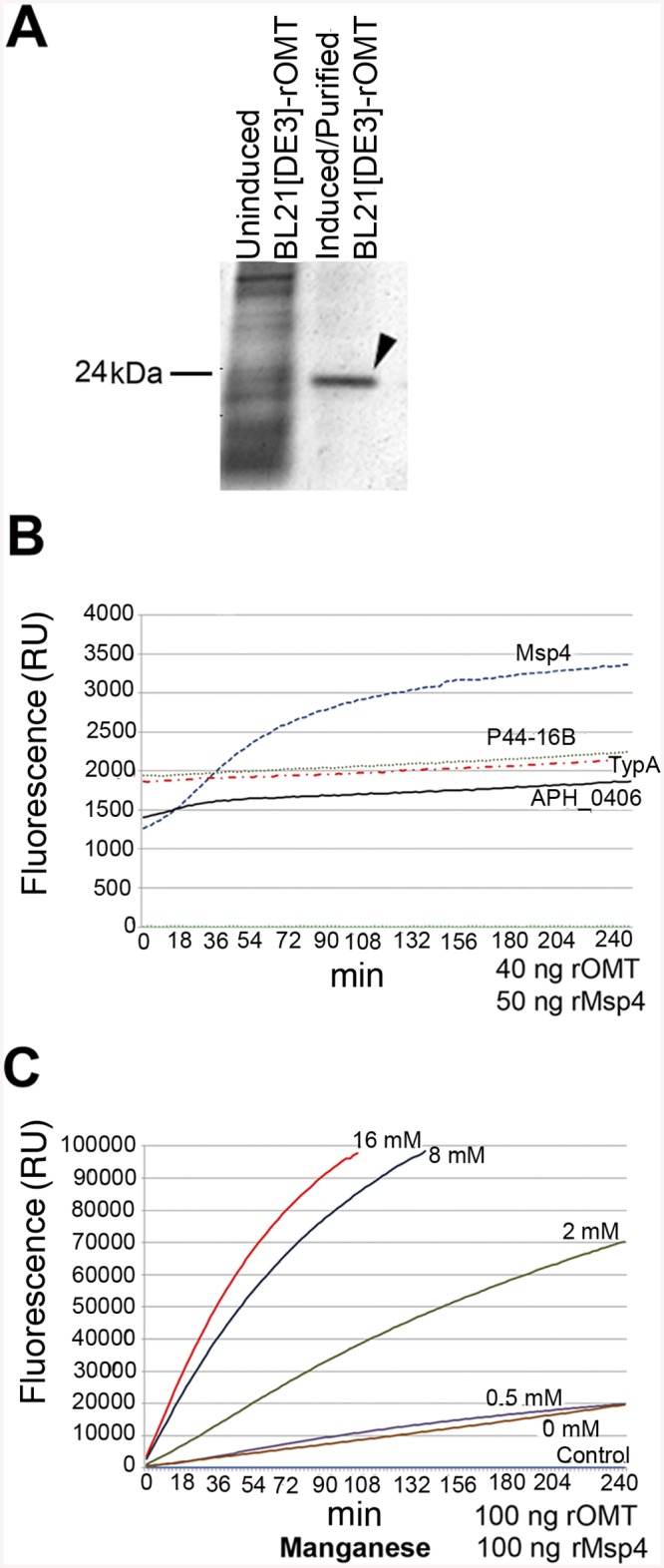
*In vitro* methylation of recombinant *A*. *phagocytophilum* proteins by rOMT and catalytic effect of metal ions. A recombinant version of the complete OMT was produced in *E*. *coli* Rosetta 2(DE3) pLysS and purified by column affinity chromatography. The eluted rOMT was visualized by Coomassie blue staining after electrophoresis in a 4–16% gel for 1 hr. The expected size of rOMT is indicated on the left; the left lane contains lysate from uninduced *E*. *coli* cells carrying the plasmid encoding OMT. The right lane contains rOMT His-tag purified from *E*. *coli* carrying the plasmid encoding OMT, following induction with IPTG. B) Enzymatic activity of the rOMT was determined in a methylation assay that measured fluorescence resulting from production of resorufin in each sample. Average fluorescence from three replicates of each sample was plotted against assay time indicated on the X-axis. The blue dotted line represents the reaction using Msp4 as the substrate, which produced a curve expected from an enzymatic reaction. The reactions with APH_0406 (black line), TypA (dotted red line), and p44-16b (dotted gray line) as substrates produced only a minimal increase in fluorescence that was not significant. Green dotted line: negative control. C) Enzymatic activity under different concentrations of additional manganese (as MnCl_2_). (Red = 16 mM, Dark Blue = 8 mM, Green = 2 mM, Purple = 0.5 mM, Brown = 0 mM, Light Blue = Negative control) was tested to determine if Mn^2+^ (included at a concentration of 10 mM in the assay kit) was a limiting factor for the OMT. Higher concentrations of MnCl_2_ resulted in a proportional increase in velocity of the reaction, indicating that Mn^2+^ was a co-factor and required at concentrations greater than 10 mM for optimal enzyme activity. Reactions with 16 mM additional MnCl_2_ (17 mM total Mn^2+^) were completed in 90 min with fluorescence levels 25 times higher than with only 10 mM. The line graphs represent the averages from 3 replicates. Standard deviations for substrate testing ranged from 5%–10%, whereas standard deviations for Mn^2+^ assays were <5% at all concentrations used.

To test if the *A*. *phagocytophilum* proteins identified by iTRAQ as potential substrates were in fact methylated by the OMT, rOMT was produced in *E*. *coli* using the complete coding sequence of the gene (*aph_0584*) cloned into the vector pET29a. The purity of rOMT was verified by gel electrophoresis (SDS-PAGE) and Coomassie blue staining (Fig 7A), and its molecular weight (MW) corresponded to the predicted MW of ~24 kDa for OMT (Fig 7A). We used the SAM-fluoro:SAM methyltransferase Assay to measure the activity of purified rOMT in *in vitro* methylation assays with potential substrates (http://www.gbiosciences.com/ResearchProducts/samfluoro.aspx). In this assay, the production of highly fluorescent resorufin (expressed as resorufin units, RU) resulting from oxidization of 10-acetyl-3,7,- dihydroxyphenoxazine (ADHP) by hydrogen peroxide generated during the reaction and monitored at an excitation wavelength of 540 nm and an emission wavelength of 595 nm. Two higher molecular weight proteins present in the un-induced *E*. *coli* lysate co-eluted with rOMT. Methylation assays using only the rOMT along with all reagents except for the substrate (negative control) did not demonstrate any detectable increase in fluorescence in the presence of these contaminant proteins, indicating that they did not affect the results of the assay (Fig 7B).

Recombinant versions of proteins identified by iTRAQ as differentially methylated between the mutant and the wild-type bacteria were also produced in *E*. *coli*, purified as done for rOMT, and tested in the *in vitro* methylation assay. rOMT (40 ng) and four recombinant *A*. *phagocytophilum* protein substrates (Msp4, APH_0406, TypA, and P44-16b; 50 ng each) were used in methylation reactions for 4 hr at 34°C. We selected these proteins from eight candidates that yielded the strongest reduction in abundance ratios of <0.60 (Table 3). Production of recombinant preprotein translocase subunit SecA was unsuccessful in One Shot BL21[DE3] chemically competent *E*. *coli* (Invitrogen, New York), BL21[DE3] (New England Biolabs, Massachusetts), and in Rosetta 2[DE3] *E*. *coli* pLysS (Novagen, Germany), thus it was not pursued further. The number of RU (fluorescence) from known concentrations of resorufin (0 μM, 5 μM, 10 μM, 25 μM, and 50 μM) was determined to produce a standard curve, and the concentration of resorufin produced in each reaction was calculated from the standard curve values. Of the four proteins tested, only rMsp4 resulted in a significant and rapid increase in resorufin production (expressed as resorufin units, RU) when incubated with rOMT (Fig 7B). rAPH_0406, rTypA, and rp44-16b produced high background fluorescence that resulted in high initial readings (~1,500–2,000 RU; 1.6–2.1 μM), but did not continue to accumulate a significant number of RU, and only reached values of ~2,200 RU (2.3 μM) (Fig 7B). By contrast, when rMsp4 was used as the substrate, the fluorescence started at a lower reading (1,200 RU; 1.3 μM) but climbed to higher values (~3,400 RU; 3.6 μM), and reached a plateau at around 210 min after the reaction was initiated (Fig 7B). Kinetics of the enzyme reaction were tested with 60, 80, and 100 ng of enzyme with a constant concentration of 50 ng rMsp4, and in the presence of 80, 100, and 150 ng of rMsp4 with a constant concentration of rOMT at 40 ng. We expected that if Msp4 was the substrate, the velocity of the reaction would increase with increasing concentrations of the enzyme and substrate, which would result in a shorter time for the reaction to reach Vmax (the maximum initial velocity when all enzyme molecules present in the reaction are in complex with the substrate). As predicted, the reaction reached Vmax in less time with higher concentrations of rOMT or rMsp4 (S4 Fig). At the time required for the enzyme to reach Vmax, the rOMT had an activity of 0.13 μM/min with a Km of 5.57x10^5^ M and the reaction reached Vmax after 46 min of initiation (Table 4). Because of the slow reaction kinetics, we suspected that *A*. *phagocytophilum* OMT required the addition of specific metal ions to catalyze the reaction, similar other *o*-methyltransferases [40]. Several concentrations (0.5 mM, 2 mM, 8 mM, and 16 mM) of MnCl_2_ were added to the methylation reaction containing 100 ng (17.89 ρmoles) of rOMT and 100 ng (16.61 ρmoles) of rMsp4. This was in addition to the 10 mM Mn^2+^ already included in the kit (GBiosciences, pers. comm.). Addition of Mn^2+^ resulted in greater fluorescence (higher RU) (Fig 7C) and faster reaction times, reaching peak levels of RU by ~100 min after initiation. With the addition of 16 mM of MnCl_2_ (17 mM total Mn^2+^), 98,000 RU (53.8 μM) were reached compared to 20,000 RU (11 μM) when the enzyme and substrate were used alone with the 10 mM Mn^2+^ supplied in the kit (Fig 7C). Similar decreases in reaction time (130 min) were observed with the addition of 8 mM of MnCl_2_ (Fig 7C). The higher activity of the enzyme was also evident from the changes in enzyme activity (Km), and in the time to reach Vmax (1.67x10^4^, 1.77 μM/min, and 9:00 min, respectively) (Table 4). In preliminary tests, MgCl_2_ did not accelerate the activity of the enzyme reaction significantly.

**Table 4:** *In vitro* reaction kinetics of the methylation of Msp4 by OMT.

Enzyme	Km (M)	Enzymatic Activity (μM/min)	Time at Vmax (min)
**OMT**	5.57x10^5^	0.14	46:03.1
**OMT + 16 mM MnCl**_**2**_ *	1.67x10^4^	1.77	09:00.0
Positive control**	N/A	N/A	01:00.0

* Reaction buffer contains 10 mM Mn^2+^

** Adenosine Homocysteine.

### Crystal structures of the *A*. *phagocytophilum* OMT

Protein for crystallization experiments was produced and purified by Seattle Biomed, a collaborator within the Seattle Structural Genomics Center for Infectious Disease (SSGCID), and was crystallized as described in the Materials and Methods section. Although this target has 33% sequence identity to its closest neighbor in the Protein Data Base (PDB), phases for the initial X-ray data from the synchrotron could not be determined by molecular replacement (MR). We initially hypothesized that binding of a substrate or co-factor would alter the conformation of the protein to something more amenable to MR. However, even after co-crystallizing the protein with SAH, phases for the X-ray data still remained recalcitrant to being solved by MR. Therefore, we chose to pursue single wavelength anomalous diffraction (SAD) phasing by using high concentration soaks (0.5 M) with sodium iodide solution, as it has previously yielded *de novo* phases for many other targets from the SSGCID [41]. Iodide-SAD data were collected on our in-house X-ray generator (Table 5) and PHENIX HySS was able to find 72 iodide ion sites during its search, but we were able to identify 118 in the final structure using anomalous difference map peaks with a contour level of 3.5 σ. Phases for the *Apo*, SAM-Mn^2+^, and SAH-Mn^2+^ datasets (Table 5) were then determined by MR, using the SAH-bound structure as a search model.

**Table 5:** X-ray reflection and refinement statistics.

Target ID	AnphA.01233.a + SAH Iodide-SAD	AnphA.01233.a *Apo*	AnphA.01233.a + SAM + Mn	AnphA.01233.a + SAH + Mn
PDB ID	4OA5	4OA8	4PCL	4PCA
Space Group	*C*2	*P*2_1_22_1_	*P* 6_3_22	*P* 2_1_2_1_2_1_
a, b, c (Å)	192.36, 72.27, 130.84	73.15, 76.40, 79.53	123.87, 123.87, 121.31	85.21, 102.76, 103.32
a, b, g	90.0, 124.94, 90.0	90.0, 90.0, 90.0	90.0, 90.0, 120.0	90.0, 90.0, 90.0
Beamline	FR-E+ Superbright	APS LS-CAT 21ID-G	APS LS-CAT 21ID-F	APS LS-CAT 21ID-F
Wavelength (Å)	1.54	0.97856	0.9787	0.9787
Collection Temp. (K)	100	100	100	100
Resolution Range (Å)	50.0–2.30 (2.36–2.30)	50.0–2.15 (2.21–2.15)	50.0–1.85 (1.90–1.85)	50.0–1.50 (1.54–1.50)
Unique Reflections	127137 (9254)	24484 (1798)	46968 (3382)	144437 (10523)
Completeness (%)	98.8 (97.7)	98.2 (98.1)	99.3 (98.9)	99.5 (99.1)
Multiplicity	3.72 (3.56)	4.71 (4.47)	5.64 (5.71)	6.18 (6.18)
I/s(I)	16.88 (5.60)	27.49 (3.39)	18.36 (3.50)	16.27 (3.14)
R_merge_	0.072 (0.301)	0.048 (0.491)	0.064 (0.536)	0.073 (0.573)
Refinement Resolution (Å)	50.0–2.30	50.0–2.15	50.0–1.850	50.0–1.50
Reflections Used in Refinement	62137	24468	46964	144413
R_free_ Reflections	3320	1244	2358	7142
R_work_/R_free_	0.195/0.231	0.185/0.232	0.170/0.209	0.1469/0.1721
RMSD Bonds Lengths/Angles	0.011/1.458	0.015/1.597	0.011/1.234	0.016/1.694
Average Protein B-factor (Å^2^)	20.31	38.33	28.87	15.92
Average Solvent B-factor (Å^2^)	24.41	38.25	37.58	30.52
Average Ion/Ligand B-factor (Å^2^)	30.81	53.09	27.23	11.16
No. Protein Atoms	9684	3168	3340	6972
No. Solvent Atoms	849	177	408	1006
No. Ion/Ligand Atoms	334	6	56	108

**Fig 8:**
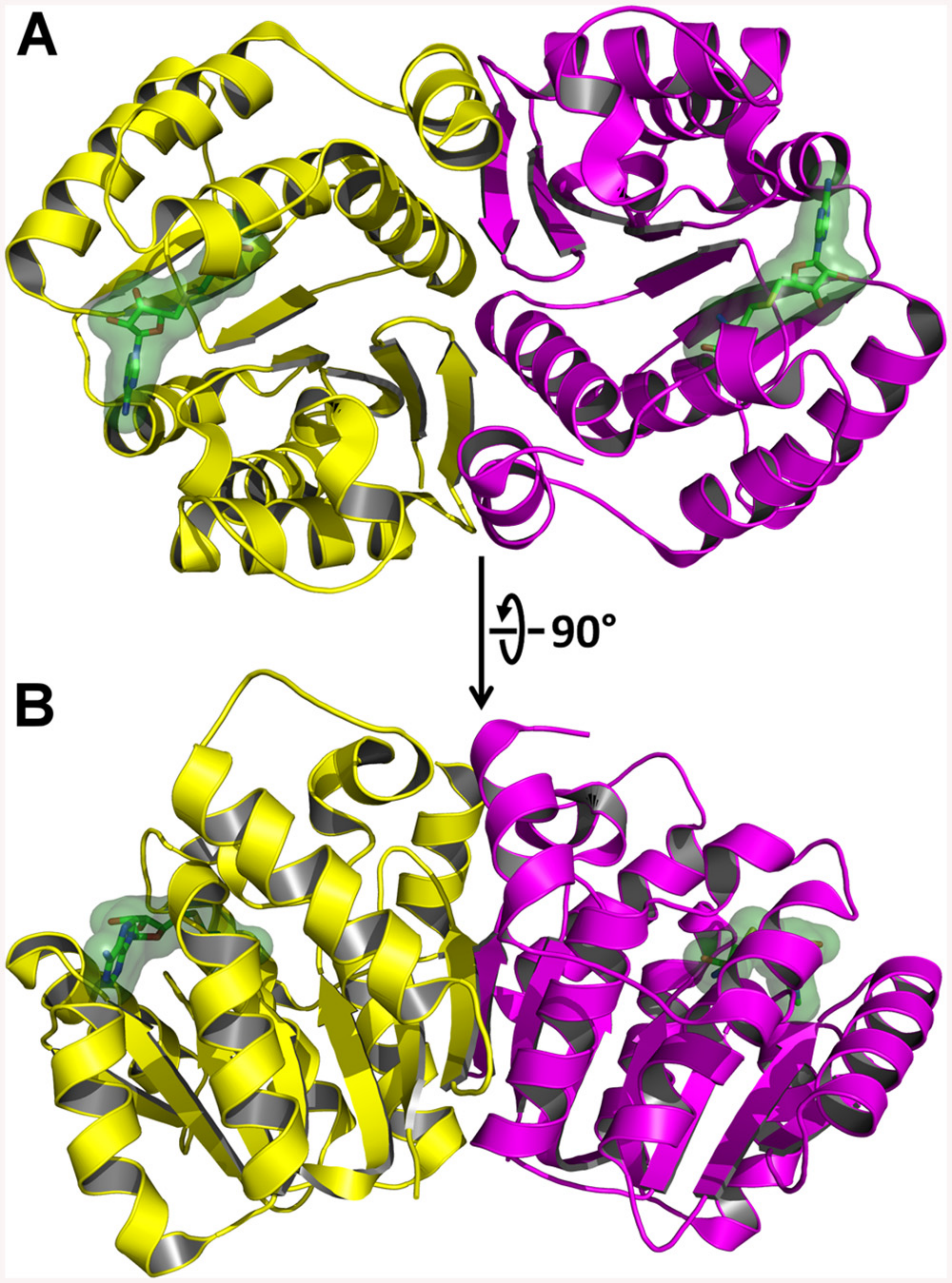
X-Ray Crystal Structure of AnphA.01233.a Bound to SAH. The crystal structure of *A*. *phagocytophilum* OMT was produced in association with SAH molecules or without (*Apo*). A) Ribbon representation of the AnphA.01233.a dimer crystal structure. Molecules A and F from the PDB 4OA5 are colored yellow and magenta, respectively. The bound SAH molecule is represented in stick format surrounded by a transparent green surface. B) View of the AnphA.01233.a dimer rotated about the X-axis by 90 degrees as compared to A.

AnphA.01233.a has a canonical *o*-methyltransferase fold which consists of a central 7-stranded β-sheet that is flanked on both sides by three α-helices (Fig 8). Since the structure was not solvable by MR, we assayed the PDB for structural homologues using the full-PDB SSM search on the PDBeFold website. The nearest homologue was 3CBG, another *o*-methyltransferase from *Cyanobacterium synechocystis*, which had a C_α_ RMSD of 1.69 Å^2^. With this much of a difference in structural similarity in the PDB, it is not surprising that MR failed to provide phases. AnphA.01233.a crystallizes as a dimer in both the *Apo* and SAH-bound crystal forms—the *Apo* form has one dimer per asymmetric unit, while the SAH-bound form has three dimers per asymmetric unit (Fig 8). The SAH molecule binds at the apex of the β-sheet, and the binding pocket is completely solvent exposed. When aligning a monomer of the *Apo*- and SAH-bound structures, the RMSD for all C_α_ carbons is only 0.273 Å^2^, so no large conformational changes occur due to ligand-binding. However, there is a small movement in a helix, composed of residues 31–40, that moves towards that substrate in the SAH-bound form as compared to the *Apo* form (S5 Fig).

**Fig 9:**
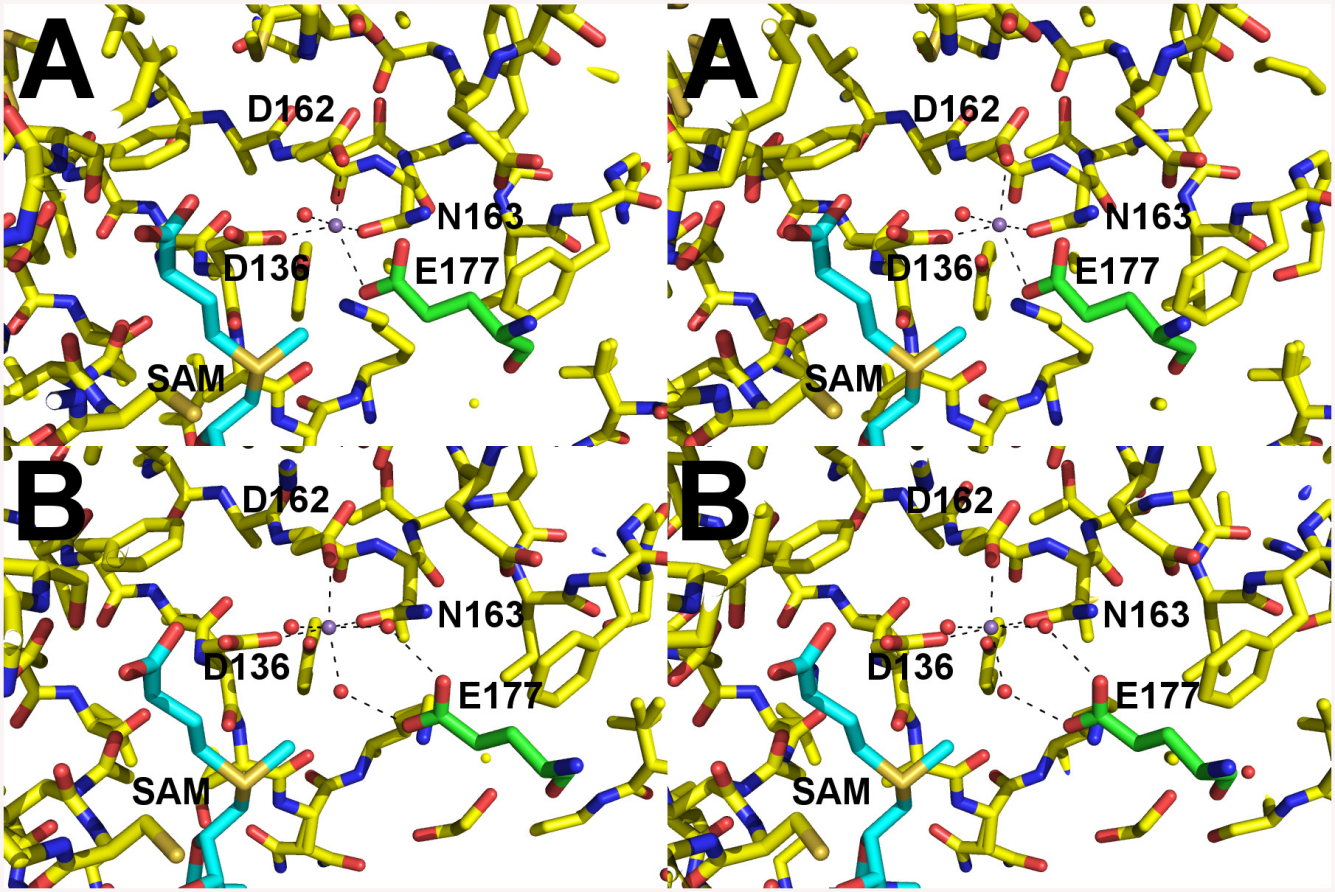
Catalytic site of AnphA.01233.a Bound to SAM and Mn^+2^. Cross-eyed stereo view of the catalytic site of AnphA.01233.a when bound to both SAM and Mn^+2^ in stick representation. Carbons are colored yellow, nitrogens blue, oxygens red, sulfurs orange. Coordinating waters and the manganese ion are shown as red and purple spheres, respectively. Carbons from E177 from the neighboring asymmetric unit are colored green for clarity. A) In chain A E177 interacts directly with the Mn^+2^. B) However, in chain B interaction with the Mn^+2^ is mediated by water molecules.

Enzymatic assays showed that the catalytic activity of the OMT was greatly increased in the presence of the divalent metal ion Mn^2+^ at >10 mM concentration. Therefore, we chose to attempt co-crystallization experiments with Mn^2+^ in the presence of both SAM and SAH. Crystals formed readily in multiple initial sparse matrix screen conditions within a week and produced higher resolution data than either of the previous datasets collected in the absence of Mn^2+^ (Table 5). After molecular replacement and initial refinement of these structures, a positive F_o_-F_c_ map peak at a contour level of 25 σ was observed in both the 4PCA and 4PCL structures, indicating that manganese was bound to the protein in close proximity to the SAH/SAM binding site (Fig 9). The Mn^2+^ ion is coordinated by the side-chains of D136, D162, and N163 and waters from the solvent (Fig 9). This places the Mn^2+^ ion within 4.5 Å of the CE methyl group to be transferred from the SAM co-factor to the hypothesized glutamate substrate of Msp4. Interestingly, a glutamic acid residue from a neighboring asymmetric unit, E177, inserts into a catalytic site in the SAM- Mn^2+^ bound structure. It appears to adopt a slightly different conformation for either chain A or chain B, which contains 2 molecules of OMT per asymmetric unit. In chain A, E177 interacts directly with the manganese ion at a distance of 2.5 Å (Fig 9A), whereas in chain B, interaction of E177 with the Mn^2+^ ion is mediated by two water molecules (Fig 9B). Since the natural substrate of this enzyme is a glutamic acid residue(s) from Msp4, it is likely that the glutamic acid from Msp4 interacts with the OMT enzyme similarly to this.

### OMT protein is present in other Anaplasmataceae but absent in members of the Rickettsiaceae

In order to understand the possible relationship of the *A*. *phagocytophilum* OMT with other members of this family of enzymes, PSI-BLAST was used to search for homologous OMTs in other organisms. Within the order *Rickettsiales*, only members of the families *Anaplasmataceae* and *Candidatus* Midichloria mitochondrii (from the new family “*Candidatus* Midichloriaceae”) encoded OMTs related to *A*. *phagocytophilum* OMT (S6A Fig). However, Δ*-proteobacteria* encoded OMTs that had even closer homology to *A*. *phagocytophilum* OMT, including OMTs from *Bdellovibrio bacteriovorus*, *Gloeocapsa sp*., *Anaeromyxobacter dehalogenans*, and *Haliangium ochraceum* (S6A Fig). A PSI-BLAST search assigned a better e-value (4e-40) to an OMT from *B*. *bacteriovorus* than to the *C*. M. mitochondrii OMT (2e-36), suggesting that the former enzyme more closely resembled *A*. *phagocytophilum* OMT. Furthermore, when the three motif sites detected by MEME (S6B Fig) from the four OMT enzymes were compared, the Δ-*proteobacteria* OMTs appeared to be more similar to *A*. *phagocytophilum* OMT than the *C*. M. mitochondrii OMT (S6B Fig). Motif 1 of the *A*. *phagocytophilum* OMT had 48% identity and 66% similarity to *H*. *ochraceum* OMT motif 1, respectively, whereas *C*. M. mitochondrii OMT motif 1 only showed 33% identity and 53% similarity. *A*. *phagocytophilum* OMT motif 2 exhibited 60% identity and 74% similarity with the corresponding motif in *B*. *bacteriovorus* OMT compared to values of 52% identity and 67% similarity to the motif regions of *C*. M. mitochondrii OMT motif 2. *A*. *phagocytophilum* motif 3 showed 44% identity and 72% similarity to *B*. *bacteriovorus* OMT motif 3 compared to 33% identity and 67% similarity with that motif in *C*. M. mitochondrii OMT (S6B Fig).

### Putative positions of methylated residues in Msp4

The tertiary structure of Msp4 was predicted using Phyre2 [42] that compares conserved residues of a query protein to the sequence of proteins with known crystallized structures. The predicted tertiary and secondary structures were used to predict the probable positions of the methylated residues. Msp4 was predicted to form a β-barrel typical of porins (S7B Fig), and the glutamic acid residues that are modified by the OMT are predicted to be located at the start of one of the β-strands forming the beta-barrel (S7A and S7B Fig). Furthermore, transmembrane and signal peptide prediction software suggested that the first ~30 aa residues represented a signal peptide to direct transport of the protein from the cytoplasm to the outer membrane (S7C Fig). These residues corresponded to the α-helix at the N-terminus (dark blue) that is probably cleaved before the protein is positioned in the outer membrane (S7B Fig). The protein does not contain predicted transmembrane domains, but it is very likely that its positioning in the outer membrane is similar to that reported for other porins in that the β-barrel spans the membrane, and the portion of the protein with the longest loops is exposed on the outside of the bacteria.

## Discussion

Genetic manipulation of *A*. *phagocytophilum* and other members of the *Anaplasmataceae* is difficult due to the intracellular nature of these organisms, and currently relies on random mutagenesis to study the role of specific genes during pathogenesis in the mammal and development in the tick [18,37,43]. Targeted mutagenesis in the related organism, *Ehrlichia chaffeensis*, proved ultimately unsuccessful as the transformants obtained were not able to persist *in vitro* for more than six days [43]. The recent success of targeted mutagenesis in *Rickettsia rickettsii* resulting in the disruption of a major surface protein gene (*ompA*) [44] presumed to be a virulence factor without producing a detectable defect provides impetus to develop this method for other *Rickettsiales*, and serves as a reminder that gene function ultimately must be confirmed by mutational analysis. In this manuscript, we report the effects of the mutation of a specific gene of *A*. *phagocytophilum* that we suspect abolished its ability to infect tick cells. However, due to the lack of a complementation system in the *Anaplasmataceae*, we cannot completely rule out that this change in phenotype was due to secondary mutations. Nevertheless, our conclusions are supported by the effect of the methylation inhibitor AdOx, which mimicked the mutation at a concentration of 30 nM (Fig 3). Previously, Chen *et al*. [45] described an *A*. *phagocytophilum* mutant with an insertion in the dihydrolipoamide dehydrogenase 1 (*lpda1*) gene at the APH_0065 locus, which altered the inflammatory response during infection of mice by increasing the production of reactive oxygen species [45], but had no effect on *in vitro* growth. The mutant (ΔOMT) described here was selected in the human cell line HL-60 in which it replicated in a manner comparable to wild-type bacteria (Fig 2). The mutant had an insertion in *aph_0584* encoding an *o*-methyltransferase (OMT) family 3 (GI: 88598384; E.C. 2.1.1.24). The inability of ΔOMT to replicate within tick cells highlighted the distinct mechanisms used by *A*. *phagocytophilum* for colonization of mammal and tick hosts.

It is interesting that the search for OMTs similar to that encoded by *aph_0584* only identified an OMT in one *Rickettsiales* organism, i.e., in *C*. M. mitochondrii, which is outside the family *Anaplasmataceae*. This intracellular organism develops in the mitochondria of *I*. *ricinus* ticks and is a member of a new family thought to be closely related to, but distinct from, the *Anaplasmataceae* [46]. Phylogenetic analysis showed highest similarity with enzymes from members of the *Δ-proteobacteria* (S6A Fig) and analysis of the different motifs present in the OMT indicated that *A*. *phagocytophilum* OMT is more similar to the OMT from the Δ*-proteobacterium B*. *bacteriovorus* than to that from *C*. M. mitochondrii (S6B Fig). *B*. *bacteriovorus* is a predatory bacterium that attacks gram-negative bacteria and, like *A*. *phagocytophilum*, presents a biphasic life cycle with an “attack form” that attaches to the host cell and a “dividing form” that occurs only in the periplasm of its host within a vacuole formed by its own proteins as well as host proteins [47]. Like *A*. *phagocytophilum*, *B*. *bacteriovorus* differentially expresses genes depending on the phase of development during infection [48]. Two OMTs are differentially regulated depending on the phase of infection; one OMT is up-regulated upon entry to Bdelloplast (Bd2861) and another extracellularly (Bd0381) [48]. Whether or not the up-regulation of the OMT in *B*. *bacteriovorus* during cell invasion is involved in the methylation of proteins important for entry is not known, however its up-regulation indicates that it may play such a role. It is possible that an ancestor of the families *Anaplasmataceae* and “*C*. Midichloriaceae” obtained their OMTs from members of the Δ*-proteobacteria* by lateral gene transfer. This possibility is supported by the absence of members of this enzyme family in the *Rickettsiales*, in which the only enzyme that showed slight similarities with the OMT was a bifunctional methyltransferase (m7G46) present in some *Rickettsia* species, albeit with high e-values of e0.28 –e1.2. These family 3 OMTs existing in *Anaplasmataceae* and the new family “*C*. Midichloriaceae” are evidently not required by other members of the *Rickettsiales* for infection of ticks, which seem to utilize different methyltransferases to carry out similar functions [23].

OMTs of the type encoded by *aph_0584* methylate free carboxyl groups on glutamic acid residues of bacterial chemoreceptors [49–51]. Thus, they are involved in environmental sensing, which could also be the case in *A*. *phagocytophilum* since a sensor-histidine kinase CckA (*aph_0582*) is predicted to localize to the membrane and to be involved in signal transduction and regulation of transcription (http://www.uniprot.org/uniprot/Q2GKC9). Notably, CckA is part of the *A*. *phagocytophilum* two component system, and is paired with response/regulator transcription factor CtrA, allowing *A*. *phagocytophilum* to respond to environmental changes [52]. A lack in the ability of the ΔOMT to respond when transferred from mammalian to tick cells could explain the continued expression of a set of proteins known to be important for infection of mammalian cells, and to be down-regulated in tick cell culture (Table 1; S3 Fig) [17,38]. However, it is unlikely that these phenotypic changes are due to a polar effect on the expression of the sensor-histidine kinase (*aph_0582*) since the transposon promoter drives transcription in the opposite direction from *aph_0582* and the insert is located at a distance of around 1600 bp from that gene (Fig 1B). Furthermore, transcription of the flanking genes is independent of the expression of the *omt* gene, and they do not appear to be part of an operon since no bands were amplified from the intergenic regions between these genes (S8 Fig). Although methyltransferases modifying glutamic acid residues have recently been identified as being widely conserved in eukaryotes, their targets, poly(A)-binding proteins, are methylated at additional amino acid residues, placing these enzymes in a different class from *A*. *phagocytophilum* OMT [53]. Interestingly, the OMT from *B*. *bacteriovorus* (Bd0381) which is homologous to *A*. *phagocytophilum* OMT (e-value 1e-42) was shown to be up-regulated when the bacteria were extracellular along with several genes involved in chemotaxis and sensing, including a methyl chemotaxis protein (Bd2503), pilS sensor protein (Bd1512), two-component response regulator (Bd0299), and a sensory box histidine kinase (Bd1657) [48]. Whether the *B*. *bacteriovorus* OMT, Bd0381, is involved in the methylation of the methyl chemotaxis protein is not known. However, it is possible that this OMT plays a role in environmental sensing, and that acquisition of a gene encoding such an enzyme enabled members of the *Anaplasmataceae* family to adapt to environmental changes more efficiently.

Analysis of the behavior of ΔOMT in ISE6 cells demonstrated reduced binding of *A*. *phagocytophilum* to tick cells (Fig 3A), partly explaining the decrease in bacterial numbers seen as early as 1 day p.i. (Fig 2). However, binding was not completely abolished by either the mutation or treatment with Adox (Fig 3). Therefore, we investigated differences in bacterial internalization and intracellular development. Although the ΔOMT that did bind to tick cells were readily internalized between 1–20 hr (Fig 6A and 6B), the bacteria were not able to form morulae and replicate intracellularly, but persisted as individual bacteria within ISE6 cells for at least 10 days p.i. (Fig 6C–6H). Furthermore, we verified that the ΔOMT bacteria were internalized within ISE6 cells by confocal microscopy and a trypsin-protection assay (S2 Fig). Methylation of outer membrane proteins and virulence factors is increasingly recognized as an essential process during host invasion and infection by several obligately and facultatively intracellular bacteria [22,28,30,32,54–58]. Therefore, we considered that the lack of methylation of glutamic acid residues in Msp4 may have played a role in the reduced adhesion to tick cells and was probably responsible for preventing replication of ΔOMT in tick cell culture. Methylation of proteins that mediate adhesion to host cells has been reported in other members of the *Rickettsiales* [54,59]. Methylation of *R*. *prowazekii* OmpB by lysine methyltransferases was shown to play a role in adhesion to and infection of endothelial cells, and to be important for virulence [59]. Nevertheless, *E*. *coli* expressing recombinant OmpB were able to bind to endothelial cells in the absence of methylation [31]. Likewise, *E*. *coli* transformed to express recombinant Msp4 were able to adhere to ISE6 cells in the absence of the *omt* gene (S9A Fig and S5 Text). Furthermore, *E*. *coli* transfected only with *msp4* construct bound more readily than those harboring both *msp4* and *omt* (S9A Fig). Analysis of the OMT protein sequence using Phobius predicted a non-cytoplasmic location of the enzyme, suggesting that methylation of Msp4 occurred in the periplasm of *A*. *phagocytophilum* (S9B Fig). It is likely that OMT is not transported to the periplasm in *E*. *coli* and methylation is thus not carried out efficiently. Because *E*. *coli* transfected with only *msp4* were able to bind to tick cells, methylation of the protein was not essential for adhesion, explaining why disruption of *omt* only reduced but did not abolish *A*. *phagocytophilum* binding to tick cells (Fig 3). Productive infection of cells by *A*. *phagocytophilum* requires completion of a multi-step process for efficient invasion and replication to occur. Increased expression of OMT in bacteria bound to ISE6 cells compared to those adhering to HL-60 cells (Fig 5) suggested that physical contact with the tick cell outer membrane induced OMT expression. Induction of OMT expression happened rapidly, and waned as bacteria passed into the cytoplasm.

iTRAQ identified several potential substrates of the enzyme in *A*. *phagocytophilum* and *I*. *scapularis* cells, two of which included *A*. *phagocytophilum* proteins previously shown to be highly expressed during infection of ISE6 cells, i.e., Msp4 and APH_0406 [17]. However, *in vitro* methylation assays (Fig 7B and 7C) using recombinant versions of potential substrates only confirmed Msp4 (Fig 7B), and identified Mn^2+^ as the most effective cofactor, indicating that *A*. *phagocytophilum* OMT is a metal dependent methyltransferase. The kinetics of the reaction were comparable to those reported for methyltransferases from *R*. *prowazekii* and *R*. *typhi* in which the linear portion of the reaction curve occupied 50–300 min [30,54]. It is possible that the substrate protein, Msp4, is methylated by OMT as a linear molecule prior to translocation rather than as a folded protein, as used here, and this may further explain the slow *in vitro* assay kinetics. These results confirmed Msp4 as a substrate of the OMT, but whether the enzyme methylates other proteins awaits further investigation. The other proteins that showed differential methylation in the proteomic analysis, but were not methylated *in vitro*, are possibly methylated by other methyltransferases present in *A*. *phagocytophilum* or in the host cell.

Msp4 is an antigenic protein encoded by a single copy gene that is highly conserved between different strains of *A*. *phagocytophilum* [60], as well as in other members of the genus *Anaplasma* [61]. As a member of the Msp2 superfamily of proteins [62], Msp4 is homologous to *A*. *phagocytophilum* Msp2 (P44), which has been shown to facilitate binding to mammalian cells, to be a porin and to be post-translationally modified. It is likely that Msp4 and Msp2 (P44) are structurally and functionally similar but that they have evolved to function in the tick vector and mammal, respectively. The most common non-specific bacterial porins form 16-strand β-barrels configd as trimeric peptide subunits [63,64]. More substrate specific bacterial porins are comprised of 18-, 14-, 12-, or 8-strand β-barrels and in some cases are present as monomers (e.g., the 14 beta-stranded porins OmpG and CymA in *Escherichia coli*) [63]. Since Msp4 is predicted to contain a 14-stranded β-barrel (S7 Fig), and by homology with the *A*. *marginale* Msp4 is likely monomeric [65], we conclude that it is probably substrate specific, which is supported by its activity exclusively in tick cells. It is interesting to note that the glutamic acid residues (E) of Msp4 that appear to be important for *A*. *phagocytophilum* development inside tick cells are close to one of the loops on the outside of the channel (S7 Fig). Similarly, *L*. *interrogans* OmpL32 contains methylated glutamic acid residues that are important for infection and colonization of kidney and liver cells [32]. However, more research is needed to determine the exact function of these methyl modifications of Msp4. We realize that structure predictions can be unreliable, and ideally the crystal structure of Msp4 in association with that of OMT should be resolved. We also expect that an *A*. *phagocytophilum msp4* mutant would display a similar or even more severely compromised phenotype than the *omt* mutant, since it is possible that other enzymes participate in methylation of the Msp4. Until such a mutant is available, the predictions serve as a starting point to infer potential implications of this modification for the biology of this bacterium.

The change in phenotype and our proteomic analysis support the conclusion that methylation of Msp4 may be necessary for efficient and productive infection of tick cells. Some porins have been shown to display double functionality, acting also as adhesins and being expressed under specific environmental conditions [64], characteristics that could fit Msp4 [63]. Partial inhibition of adhesion due to the mutation of *omt* is not surprising, as it is likely that more than one adhesin is involved in binding to tick cells. This has been shown for invasion of mammalian cells by *A*. *phagocytophilum*, where three adhesins have been identified, OmpA, Asp14 and AipA [66–68]. *Rickettsiaceae* possess two additional adhesins besides OmpB and OmpA, named Adr1 and Adr2. These were recently identified by proteomic approaches and also presented putative β-barrel structures [69]. Adhesins may also serve to protect the bacteria from mammalian complement that is abundantly present in the blood, and consequently ingested with the tick blood meal. *Rickettsia conorii* OmpB β-peptide has been shown to interact with mammalian complement regulatory factor H via the exposed loops extending from the transmembrane β-barrel structure, and a number of bacterial factor H-binding proteins have been identified as adhesins [63,64]. *Anaplasma phagocytophilum* also evades complement-mediated killing, but it is not known whether this capability is mediated by binding of complement regulatory factors, or by direct interaction with complement [65]. In the cell-culture system used here, such factors would not be relevant due to the absence of active complement.

The most significant phenotypic change due to the mutation in the *omt* gene was the inability of *A*. *phagocytophilum* to replicate and form normal morulae within ISE6 cells (Fig 6C–6H). In ISE6 cells, ΔOMT persisted as individual bacteria, while wild-type bacteria formed large morulae that are distinguishable on day 3 p.i. (Fig 6D). Up-regulation of OMT expression during interaction of *A*. *phagocytophilum* wild-type with ISE6 cells (Figs 4 and 5), as compared to the inability of the ΔOMT bacteria to change protein expression (Table 1 and S3 Fig), indicated that this was necessary for normal morphogenesis of *A*. *phagocytophilum* in tick cells (Fig 6).

## Materials and Methods

### Identification and growth of the mutant

The *A*. *phagocytophilum* isolate HZ, which was originally cultured from a New York patient [70], was cultivated in HL-60 cells maintained in RPMI 1640 medium (Lifetechnologies, New York) supplemented with 10% FBS (BenchMark, Gemini Bioproducts, California), and 2mM glutamine at 37°C with 5% CO_2_ in humidified air [71]. Several transformants were generated to express Green Fluorescent Protein (GFPuv) and were selected and maintained in HL-60 cells as described [18,71]. The ability of the transformants to grow in ISE6, an *I*. *scapularis* embryonic cell line, was tested by inoculating purified cell-free bacteria or whole infected HL-60 cells into 25-cm^2^ flasks containing confluent cell layers of ISE6 cells. Cultures were maintained in L-15C300 supplemented as described, and the pH was adjusted to 7.5–7.7 with sterile 1N NaOH [18,72]. Growth and development of transformants was evaluated by fluorescence microscopy using an inverted Nikon Diaphot microscope (Nikon, New York) to detect *A*. *phagocytophilum* expressing GFPuv [73] and by examination of Giemsa stained cell samples spun onto slides.

A transposon mutant (ΔOMT) deficient for growth in ISE6 cells was cultivated in HL-60 cells as described above by passing 3 x 10^3^ infected HL-60 cells into a new flask containing 3–4 x 10^5^ uninfected cells and 20 ml of fresh medium every 5 days. Spectinomycin and streptomycin (100 μg/ml each) were added to the cultures for selection of mutants carrying the *aadA* resistance gene encoded on the transposon. The number of insertion sites in the mutant population was determined by Southern blots of DNA purified from a 25-cm^2^ flask of infected HL-60 cells, using the Puregene Core Kit A (Qiagen, Maryland) with an additional phenol-chloroform extraction step, and Phase Lock Gel Heavy (5 Prime, Maryland) to separate phases. DNA concentration was measured with a BioPhotometer (Eppendorf, New York), and DNA extracted from HZ wild-type bacteria served as control. DNA (100 ng) from the mutant and wild-type HZ was digested with *Bgl*II and *EcoR*V and samples were electrophoresed in 1% agarose gels. DNA was transferred and probed as described [73], using digoxigenin-labeled probes specific for *gfp*_*uv*_ (PCR DIG Probe Synthesis kit; Roche, Indiana). A plasmid construct, pHIMAR1-UV-SS, encoding the transposon, served as positive control [18].

To determine genomic insertion sites in the mutant population, 5 μg of ΔOMT DNA was digested with *Bgl*II, treated with DNA clean & concentrator (Zymo Research, California) and ligated into the pMOD plasmid for electroporation into ElectroMAX DH5α cells (Invitrogen, New York). ElectroMAX DH5α cells containing the transposon were selected on YT plates with 50 μg/ml of spectinomycin and streptomycin. DNA was purified by phenol/chloroform extraction and then sequenced at the BioMedical Genomics Center (University of Minnesota).

### Effects of the mutation on bacterial growth and binding

The ΔOMT and wild-type strains were grown in 25-cm^2^ flasks containing HL-60 cells as described above. Bacteria were purified from four flasks containing 25 ml of a >90% infected cell suspension by passing the cell suspension through a bent 27 G needle and filtration of the lysate through a 2 μm pore size filter. Purified bacteria were transferred to two 15 ml tubes, centrifuged at 10,000 x g for 5 min and then resuspended in 3 ml of RPMI medium supplemented as described above. Bacteria were diluted 1:40, 1:100, and 1:400 in 20 ml of uninfected HL-60 cultures, and incubated at 37°C as described above for infected HL-60 cells. Samples of 1.5 ml were taken from each culture every day for a 5-day period, and DNA was extracted as described for DNA samples used in Southern blots. The experiment was repeated in triplicate.

To generate growth curves of ΔOMT and wild-type bacteria in ISE6 cell cultures, bacteria were purified as described above, and centrifuged at 10,000 x g for 5 min at 4°C. Supernatant was discarded, and cell free bacteria were diluted in supplemented L15C300 at ratios of 1:6, 1:12, and 1:24. The experiment was done in triplicate. To assess bacterial growth, DNA was extracted from 1.5 ml of mutant and wild-type cultures of bacteria grown in ISE6 cells every 3 days for 12 days as described before.

The number of bacteria per sample was determined by qPCR using the primers msp5 fwd and msp5 rev (S1 Table) that amplify a fragment of the single copy number *msp5* gene. qPCR reactions were performed in an Mx3005p (Agilent, California) cycler, using Brilliant II SYBR Green Low ROX QPCR Master Mix (Agilent, California) under the following conditions: an initial cycle of 10 min at 95°C, 40 cycles of 30 sec at 95°C, 1 min at 50°C, and 1 min at 72°C, and a final cycle of 1 min at 95°C, 30 sec at 50°C, and 30 sec at 95°C. A standard curve was generated using the *msp5* fragment cloned into the pCR4-TOPO vector (Invitrogen, New York).

To examine binding of ΔOMT and wild-type *A*. *phagocytophilum* to tick cells, we used two different assays to evaluate adhesion to tick cells and subsequent intracellular growth and development. The first assay was carried out under stringent conditions with a low MOI that permitted sensitive assessment of the effect of the mutated *omt* gene. The second assay (described further below, under “***ISE6 infection time point experiment to evaluate intracellular development of the ΔOMT”***) was designed to allow maximal, saturating binding so that mutant bacteria could be readily observed inside tick cells. For the first assay, bacteria were purified from 20 ml of one fully infected HL-60 culture and were added to about 2.5x10^5^ ISE6 cells in 50 μl of supplemented L15C300 medium in a 0.5 mL centrifuge tube. To ensure that only activity induced during binding and not cell entry was measured, bacteria were incubated with host cells for 30 min at room temperature, flicking the tube every 5 min to enhance contact between bacteria and cells. The cells were washed twice in unsupplemented L15C300 and centrifuged at 300 x g for 5 min to remove unbound bacteria. The cell pellet was resuspended in phosphate buffered saline (PBS) and spun onto microscope slides for 5 min at 60 x g, using a Cytospin 4 centrifuge (Thermo Shandon, Pennsylvania). Slides were fixed in absolute methanol for 5 min and dried at 50°C for 10 min. Bound bacteria were labeled using an IFA with dog polyclonal antibody against *A*. *phagocytophilum* and FITC-labeled anti-dog secondary antibodies. DAPI was used to stain the host cell nuclei, and aid in host cell visualization. The number of bacteria bound to 300 cells was counted for each sample. This was repeated in triplicate, and differences were evaluated using Student’s t-test to assess significance with SigmaStats (Systat Software, California).

### Inhibition of SAM dependent methyltransferases and effect on the binding of *A*. *phagocytophilum* to ISE6 cells

To verify that lack of methylation of substrate brought about by the disruption of the *omt* gene was the cause of reduced binding of the mutant to ISE6 cells, we added adenosine dialdehyde (Adenosine periodate Oxidized, or AdOx (Sigma Aldrich, Missouri)), which inhibits SAM-dependent methyltransferases by increasing the concentration of S-adenosyl-L-homocysteine [29], to wild-type cultures. Wild-type and ΔOMT bacteria were purified from 50 ml infected HL-60 cells as described. Purified wild-type bacteria were incubated with AdOx at 20 nM, 30 nM, and 40 nM final concentrations for 1 hr at 34°C before adding them to 2x10^5^ uninfected ISE6 cells. Controls consisted of wild-type and ΔOMT bacteria purified the same way, but incubated at 34°C for 1 hr without addition AdOx.

The bacteria and ISE6 cells were incubated in 200 μl of supplemented L15C300 medium at 34°C for an additional hr to allow binding. Cells were washed 3 times in supplemented L15C300 medium by centrifugation at 600 xg to remove unbound bacteria. After the final wash, cells were resuspended in 1 ml of supplemented L15C300 medium and 50 μl of the suspension was spun onto microscope slides as described above and fixed in methanol for 10 min. The samples were incubated with dog anti *A*. *phagocytophilum* serum and labeled with FITC conjugated anti-dog antibodies. Samples were mounted in Vectashield Mounting medium containing DAPI to aid in host cell visualization (Vector Laboratories, California), and observed using a 100 x oil immersion objective on a Nikon Eclipse E400 microscope. Bacteria were counted as described above for regular binding assays, and differences in the number of bacteria per cell were evaluated using the Student-Newman-Keuls one-way ANOVA on ranks, using SigmaStat.

### ISE6 infection time course experiment to evaluate intracellular development of the ΔOMT

Two thousand LifeAct-mCherry expressing ISE6 cells [74]were seeded onto the glass portion of MatTek dishes (Ashland, Massachusetts) in 250 μl of medium the day before bacterial challenge. ΔOMT and HZ wild-type bacteria were cultured in five million HL-60 cells until > 95% infected, and bacteria were beginning to be released from cells. Cell-free bacteria were prepared by passing the infected cells through a 25 G needle 5 times, and intact cells were removed by centrifugation at 1,110 x g for 5 min. The supernatant was passed through a 2 μm pore size syringe filter to remove cell debris, and the bacteria were collected by centrifugation at 11,000 x g for 5 min. Bacteria were resuspended in 100 μl fresh culture medium and 20 μl of the suspension was inoculated onto ISE6 cell layers after 170 μL of culture medium had been removed. This resulted in a multiplicity of infection of 100–300 bacteria/cell. Dishes were incubated at 34°C in a water saturated atmosphere of 3% CO_2_ in air for 1 hr with agitation at 10-min intervals to ensure uniform exposure of cells to bacteria. Unbound bacteria were removed by washing the cells once with 2 ml of medium, and 2 mL fresh medium was added and the cultures returned to the incubator. At each of eight time points (0, 1, 2, 3, 4, 5, 7, and 10 days), culture medium was removed from MatTek dishes and cells were immediately fixed by flooding with 2 mL methanol (1 min) followed by two additional rinses (1 min) with fresh methanol. Cells were then air dried and stored at room temperature until the bacteria were labeled by IFA.

Cells were blocked with 50% FBS in culture medium for 1 hr at room temperature. Bacteria were labeled with dog anti-*A*. *phagocytophilum* serum for 1 hr followed by FITC-labeled anti-dog antibodies for 1 hr (each diluted 1:1,000). After each antibody exposure, cells were washed three times in PBS. Cells were mounted in VectaShield (Vector Laboratories) medium with 4',6-diamidino-2-phenylindole (DAPI), and examined and photographed under a cover slip using confocal microscopy as described above.

### Confirmation that ΔOMT enters ISE6 cells

LifeAct-mCherry-expressing ISE6 cells cultured in MatTek dishes were challenged with ΔOMT or HZ wild-type bacteria at a high MOI as described above, and then exposed to trypsin to confirm the confocal microscopy finding that the ΔOMT bacteria were located inside the ISE6 cells. Four days following challenge, cells were mechanically dislodged using a cell scraper, spun onto slides, air-dried and fixed in methanol. These samples represented non-trypsin controls. Cells in remaining dishes were suspended in 1 ml 0.25% trypsin-EDTA (Gibco) for 3 min, collected by centrifugation (350 x g, 2 min), resuspended in 1 ml culture medium, and 100 μl volumes were spun onto microscope slides. Remaining cells were trypsinized a second time for 2 min, washed in medium and spun onto slides. Samples (mechanically scraped cells, trypsinized 1x, trypsinized 2x) were immunofluorescence-labeled as described, mounted in VectaShield without DAPI and imaged as above (S2A–S2F Fig). As a positive control to demonstrate that trypsin removed wild-type HZ adherent to ISE6 cells, the same trypsinization procedure was followed after exposing ISE6 cells (not expressing LifeAct-mCherry) to HL60-grown HZ bacteria for one hour, and mounting slides with DAPI (S2G and S2H Fig).

### Relative omt expression measured by qRT-PCR

Relative expression of *omt* was measured at different times to determine when it was upregulated. Wild-type *A*. *phagocytophilum* HZ was purified from HL-60 cells and inoculated into four 25-cm^2^ flasks containing either 5 ml of uninfected ISE6 or 2 ml of HL-60 cultures. Bacteria inoculated into HL-60 cell cultures or ISE6 cells were incubated for 30, 60, 120, or 240 min at 37°C or 34°C, respectively. Total RNA was extracted from whole infected cell cultures, using the Absolutely RNA Miniprep Kit (Agilent, California) according to the manufacturer’s specifications. RNA was DNAse treated with 0.5 units TURBO DNAse (Ambion, New York) at 37°C for 30 min. The DNAse treatment was repeated twice and RNA concentrations were measured using a Biophotometer.

*Omt* expression was normalized against expression of the *rpoB* and *msp5* genes that had been found to be consistently expressed in both cell types during tiling array analysis [17]. qRT-PCR reactions were carried out using Brilliant II QRT-PCR SYBR Green Low ROX Master Mix (Agilent, California) using primers listed in S1 Table. Reaction parameters were as follow: one cycle at 50°C for 30 min, one denaturing cycle at 95°C for 10 min, 40 cycles that consisted of 30 sec at 95°C, 1 min at 50°C, and 1 min at 72°C, and a final cycle of 1 min at 95°C and 30 sec at 50°C. Ct values were established during amplification and the dissociation curve was determined during the final denaturation cycle. Expression of the *omt* gene was analyzed using the 2^-ΔΔct^ method, and significant differences were determined using Student’s t-test with SigmaStat.

The relative expression of the genes that resulted in a greater abundance of encoded proteins in ΔOMT (Table 1), as well as the expression of *msp4* was determined by qRT-PCR, using the primers listed in S1 Table. Total RNA was purified from 8x10^5^ HL-60 cells fully infected with wild-type or mutant bacteria, and from 8.4x10^5^ ISE6 cells fully infected with wild-type bacteria, using the Absolutely RNA Miniprep Kit. qRT-PCR reactions were carried out as described above with *msp5* and *23s rRNA* used as normalizer genes. Gene expression was determined as described above.

### Recombinant OMT production

Recombinant OMT (rOMT) protein for antibody production was produced using the pET29a expression vector (Novagen, Germany) by amplifying the entire coding region with the primers rOMT fw and rOMT rv (S2 Table) and *pfu* DNA polymerase (Promega, Wisconsin). Conditions were as follows: one denaturing cycle at 94°C for 3 min, 10 cycles with a denaturing step at 94°C for 1 min, 40°C for 1 min for annealing, and extension at 72°C for 2 min, then 20 additional cycles with a denaturing step at 94°C for 1 min, 47°C for 1 min for annealing, and extension at 72°C for 2 min, and a final extension step of 5 min at 72°C. The amplified product was digested with the restriction enzymes *Sal*I and *Eco*RV, followed by ligation into the vector at 15°C overnight. The plasmid was cloned into One Shot TOP10 competent cells (Invitrogen, New York) for replication and purified using the High Pure plasmid isolation kit (Roche, Indiana). Integrity of the plasmid was checked by sequencing with the T7 promoter (5’- TAA TAC GAC TCA CTA TAG GG– 3’) and the T7 terminator (5’- GCT AGT TAT TGC TCA GCG G– 3’) primers at the Biomedical Genomics Center of the University of Minnesota. Plasmids were transfected into BL21(D3) *E*. *coli* (New England Biolabs, Massachussets) for expression. BL21(D3) *E*. *coli* were inoculated into 100 ml of Superior Broth (AthenaES, Maryland), induced with 200 μM IPTG, and incubated at 37°C overnight with constant shaking. Protein was purified using Ni-NTA Fast Start Kit columns (Qiagen, Maryland). Protein concentrations were measured using the BCA protein assay kit (Pierce, Illinois).

Functional rOMT for enzyme activity assays was produced using the expression vector pET29a as described above but amplifying the entire open reading frame of the gene with the primers rOMTns Fw and rOMTns Rv (S2 Table), using the following conditions: one denaturing cycle at 94°C for 3 min, 10 cycles with a denaturing step at 94°C for 1 min, 45°C for 1 min for annealing, and extension at 72°C for 2 min, then 20 additional cycles with a denaturing step at 94°C for 1 min, 54°C for 1 min for annealing, and extension at 72°C for 2 min, and a final extension step of 5 min at 72°C. The amplified product was digested with *Nde*I and *Eco*R1, and ligated into pET29a to produce rOMTns lacking the S-tag present in the plasmid, and cloned into One Shot TOP10 competent cells. After confirming integrity, the plasmid was cloned into Rosetta 2(DE3) pLysS *E*. *coli* (Novagen, Germany). Transformed *E*. *coli* were incubated in 100 ml of Superior Broth and induced with 1 mM IPTG at 37°C for 5 hr. Proteins were purified with Ni-NTA Fast Start Kit columns, and protein concentration was measured as described above.

### rOMT polyclonal antibodies production

Recombinant non-functional rOMT was purified by dialysis in a 3 ml Slide-A-Lyzer Dialysis Cassette 10K MWCO (Thermo Scientific, Illinois) against tris-buffered saline (TBS), over night with one buffer change after 2 hr. Four 6–8 weeks old, female C57BL/6J mice (Jackson Laboratories, Maine) were immunized by subcutaneous (s.c.) injection of 100 μg of recombinant rOMT in TiterMax Research adjuvant (CytRx Co., Georgia). Mice were boosted twice with 100 μg of rOMT without adjuvant at 14 days and 24 days later. To obtain antiserum, blood was collected 10 days after the second booster, and unimmunized mice from the same cohort were used as controls. Serum was frozen at -20°C for future use.

### Localization assays

Wild-type bacteria were grown in 50 ml of RPMI1640 containing HL-60 cells until > 90% of the cells were infected. Around 5 x 10^7^ infected cells were used to obtain cell free bacteria by vortexing the infected cells with 60/90 grit silicon carbide (Lortone, Inc., Mukilteo, Washington) for 30 sec followed by filtration through a 2.0 μm pore size filter and centrifugation at 700 x g for 5 min to remove remaining cell debris. The percent of infected cells in the culture was calculated by counting the number of infected and uninfected cells in duplicates Giemsa-stained preparations from the same flask and the total number of cells was determined using a hemocytometer. Cell-free bacteria were incubated for 2 hr at 34°C with 2.5 x 10^5^ ISE6 cells in MatTek chambers (MatTek Corp., Massachusetts) to allow binding and expression of OMT. Unbound bacteria were removed by rinsing cells twice with culture medium. Likewise, bacteria were incubated with 2.5 x 10^5^ HL-60 cells suspended in 500 μl of culture medium for 2 hr at 37°C. Unbound bacteria were removed by washing cells twice and expression of OMT in bound bacteria was analyzed by IFA.

Cell samples were fixed for 10 min in methanol, and incubated with anti-rOMT serum diluted 1:200 in PBS containing 3% bovine serum albumin (BSA) for 2.5 hr at room temperature. Bacteria were labeled with anti-*A*. *phagocytophilum* dog serum (diluted 1:1,000). The slides were washed 3 times in PBS and blocked in PBS with 3% BSA for 10 min at room temperature. OMT expressing bacteria were then labeled with anti-mouse antibodies conjugated with AlexaFluor647 (1:500 dilution) (Jackson ImmunoResearch Laboratories, Inc, Pennsylvania) for 1 hr at room temperature. All *A*. *phagocytophilum* were labeled with anti-*A*. *phagocytophilum* dog serum diluted 1:500 followed by incubation with anti-dog IgG conjugated to FITC, using the same procedure. Tick and HL-60 cell nuclei were labeled using DAPI present in the VectaShield mounting medium. Microscopic images were obtained using an Olympus BX61 disk-scanning unit confocal microscope (Olympus America, Pennsylvania) utilizing a DSU-D2 confocal disk. Confocal images were acquired with a Photometrics Quantem:512SC EMCCD camera (Photometrics, Arizona), and high resolution images were acquired with a QFire color camera (Qimaging, California). Image capture software was Metamorph (Molecular Devices, California). ImageJ (US National Institutes of Health) was used to compile z-projections and Photoshop (Adobe Systems, California) was used for cropping.

### Measurement of differential expression of proteins and determination of methylation

HZ wild-type and ΔOMT bacteria were grown in two 50 ml volumes of RPMI1640 medium with HL-60 cells each and the number of infected cells was determined. Host cell free mutant and wild-type bacteria were purified from 2.5 x10^9^ infected cells that were ruptured by repeated passage through a 27 G needle, centrifuged at 600 x g to remove cell debris, and inoculated into a 25-cm^2^ flask containing 6 x 10^7^ ISE6 cells. This procedure was replicated three times in three independent biological replicates. Cells and bacteria were incubated for 4 hr at 34°C without agitation to allow methylation of possible substrates to be completed, since the time between up-regulation of the gene and completion of the enzymatic reaction was not known. Bacteria were then purified from ISE6 cells as described above. Bacteria were washed in unsupplemented L15C300 medium three times by centrifugation at 16,000 xg for 5 min at 4°C to remove FBS, and the final bacteria pellet was extracted for mass spectrometry.

### Protein extraction and preparation

Protein concentrations were determined by Bradford assay using two aliquots for each sample. All samples were prepared as follows at the Center for Mass Spectrometry and Proteomics at the University of Minnesota: cell pellets were reconstituted with 120 μl of protein extraction buffer [7 M urea, 2 M thiourea, 0.4 M triethylammonium bicarbonate (TEAB) pH 8.5, 20% methanol and 4 mM tris(2-carboxyethyl)phosphine (TCEP)] while on ice. Samples were sonicated at 30% amplitude for 7 sec with a Branson Digital Sonifier 250 (Emerson, Connecticut). The samples were homogenized in a Barocycler NEP2320 (Pressure Biosciences, Inc., Massachusetts) by cycling between 35 k psi for 30 sec and 0 k psi for 15 sec for 40 cycles at 37°C. Samples were alkylated for 15 min at room temperature in 8 mM methyl methanethiosulfonate (MMTS).

In-solution proteolytic digestions were performed as follow: a 200 μg aliquot of each sample was transferred to a new 1.5 ml microfuge tube and brought to the same volume with protein extraction buffer plus 8 mM MMTS. All samples were diluted 4-fold with ultra-pure water, and trypsin (Promega, Wisconsin) was added at a 1:35 ratio of trypsin to total protein. Samples were incubated for 16 hr at 37°C after which they were frozen at -80°C for 30 min and dried in a vacuum centrifuge. Each sample was then cleaned using a 4 ml Extract Clean C18 SPE cartridge (Grace-Davidson, Illinois), and eluates were vacuum dried and resuspended in dissolution buffer (0.5M triethylammonium bicarbonate, pH 8.5) to a final 2 μg/μl concentration. For each iTRAQ 4-plex (AB Sciex, California), two 50 μg replicates for each sample were labeled with iTRAQ reagent (AB Sciex, California). After labeling, the samples were multiplexed together and vacuum-dried. The multiplexed sample was cleaned with a 4 mL Extract Clean C18 SPE cartridge (Mandel Scientific Company Inc., Guelph, Canada) and the eluate was dried *in vacuo*.

### Peptide liquid chromatography fractionation and mass spectrometry

The iTRAQ labeled samples were resuspended in Buffer A (10 mM ammonium formate pH 10 in 98:2 water:acetonitrile) and fractionated offline by high pH C18 reversed-phase (RP) chromatography [75]. A MAGIC 2002 HPLC (Michrom BioResources, Inc., California) was used with a C18 Gemini-NX column, 150 mm x 2 mm internal diameter, 5 μm particle, 110 Å pore size (Phenomenex, California). The flow rate was 150 μl/min with a gradient from 5–35% Buffer B (10 mM ammonium formate, pH 10 in 10:90 water:acetonitrile) over 60 min, followed by 35–60% over 5 min. Fractions were collected every 2 min and uv absorbances were monitored at 215 nm and 280 nm. Peptide containing fractions were divided into two equal numbered groups, labeled “early” and “late”. The first “early” fraction was concatenated with the first “late” fraction by 10 mAU volume equivalents of each fraction from uv = 215 nm and repeated until all fractions were concatenated. Concatenated samples were dried *in vacuo*, resuspended in 98:2O, H_2_O:acetonitrile, 0.1% formic acid and 1–1.5 μg aliquots were run on a Velos Orbitrap mass spectrometer (Thermo Fisher Scientific, Inc., Massachussets) as described previously [76] with the exception that the Higher-energy Collisional Dissociation (HCD) activation energy was 20 msec.

The mass spectrometer RAW data (Proteowizard files) were converted as described previously [76]. ProteinPilot 4.5 (AB Sciex, California) searches were performed against the NCBI reference sequence for the *I*. *scapularis* (taxon 6945; November 14, 2011) protein FASTA database with (20468 proteins), to which the NCBI reference sequence *A*. *phagocytophilum*, HZ (taxon 212042; November 14, 2011; 1267 proteins) and a contaminant database (thegpm.org/crap/index, 109 proteins) was appended. Search parameters were: cysteine MMTS; iTRAQ 8plex (Peptide Labeled); trypsin; instrument Orbi MS (1–3ppm) Orbi MS/MS; biological modifications ID focus; thorough search effort; and False Discovery Rate analysis (with reversed database).

### Function and pathways of differentially expressed *A*. *phagocytophilum* proteins

The putative function of differentially expressed proteins identified by iTRAQ was explored using the databases in NCBI (www.ncbi.nlm.nih.gov) to identify conserved domains, Uniprot (http://www.uniprot.org/uniprot/), EMBL-EBI (http://www.ebi.ac.uk/interpro/IEntry?ac=IPR000866), and OMA (http://omabrowser.org). Pathways which involved proteins differentially expressed during infection with the ΔOMT compared to wildtype were identified using the KEGG pathway tool (http://www.genome.jp/kegg/tool/map_pathway1.html).

### Identification of differentially methylated proteins

To identify the peptides that differed in methylation, a text file of data for all peptides was imported into Excel (Microsoft, Washington), and entered into TextWrangler (Bare Bones Software, Massachusetts), which is a text editing tool. Peptides that were less abundant in the mutant when compared to the wild-type, based on the intensities of the reporter ion signals, were selected and the intensities of spectra were visually inspected in the ProteinPilot viewer software (AB SCIEX, Massachusetts) to confirm differences.

### Production of recombinant proteins for substrate testing

A functional version of the OMT without the S-tag (rOMTns) was produced and purified. Recombinant proteins of potential substrates identified by iTRAQ were produced using the pET29a vector. Primers to amplify partial or complete coding sequences were designed with restrictions sites for *Nde*I and *Xho*I enzymes (S2 Table), and DNA amplified using *pfu* enzyme under conditions listed in Table 2. The sequence integrity of purified products was confirmed by Sanger sequencing, and DNA cloned into BL21(D3) *E*. *coli* (New England Biolabs) for expression. Proteins were produced in 150 ml of Superior Broth after induction with 200 μM IPTG and used in methylation assays described below. Protein concentrations were measured using the BCA micro protein assay kit (Pierce).

The SAM-fluoro: SAM Methyltransferase Assay (G-Biosciences, Missouri) was used to determine the activity of the enzyme. In this assay, methylation by SAM-dependent methyltransferases is correlated with the production of H_2_O_2_ which can be assessed through the production of fluorescent resorufin from 10-acetyl-3,7,-dihydroxyphenoxazine (ADHP). Resorufin production was monitored using an excitation wavelength of 530 nm and an emission wavelength of 595 nm in a Synergy H1 Hybrid microplate reader (Biotek, Vermont) during a kinetic run measuring fluorescence every two min for 4 hr at 34°C. Reactions were carried out in a 96 well EIA/RIA plate flat bottom plate (Costar, New York) covered with a MicroAmp optical adhesive film (Applied Biosystems, New York) to protect samples from evaporation. Assays were performed with 40 ng (7.14 ρmole) of the enzyme and 50 ng (8.31 ρmole) of substrates. The moles of the substrates were calculated from the theoretical molecular weight in kDa, using Zbionet (http://www.molbiol.ru/eng/scripts/01_04.html). Positive controls included the addition of AdoHcy alone or in combination with rOMTns to assay reagents provided by the manufacturer.

To determine enzyme kinetics, the concentrations of the enzyme or the substrate were increased separately. For the first set of reactions, the amount of enzyme was increased to 60 ng (10.71 ρmole), 80 ng (14.29 ρmole), and 100 ng (17.89 ρmole), while the substrate was left at 50 ng. In the second set, the enzyme concentration was left at 40 ng, while the substrate was used at 80 ng (13.29 ρmole), 100 ng (16.61 ρmole), and 150 ng (24.92 ρmole). Each reaction was done in triplicate and the average of the reactions was analyzed. To determine whether the 10 mM Mn^2+^ included in the kit was a limiting factor for rOMT activity, additional MnCl_2_ was included at 0.5 mM, 1 mM, 2 mM, 4 mM, 8 mM, and 16 mM concentrations in reactions containing 100 ng of the OMT and 100 ng of the substrate. All reactions were carried out in triplicate using the manufacturer’s recommendations, and the kinetics of the enzyme were analyzed using the Michaelis-Menten equation.

### Crystallization of *A*. *phagocytophilum* OMT

Crystals of OMT from *A*. *phagocytophilum* were obtained via the sitting drop vapor diffusion method, where 400 nl of protein solution was mixed with 400 nl of precipitant solution in the sample well and then equilibrated against 80 μl of precipitant in the reservoir well of 96-well Compact 300 crystallization plates (Rigaku Reagents, Washington). For *Apo* OMT, the protein concentration used was 20 mg/ml and the precipitant solution was 0.2 M MgCl_2_, 0.1 M TRIS at pH 8.50, and 20% PEG 8000. For SAM-Mn and SAH-Mn bound structures protein at 20 mg/ml was pre-incubated with 2 mM of either SAM or SAH and 10 mM MnCl_2_ for 1 hr before setting up trays. Final crystallization conditions for SAM-Mn^+2^ and SAH-Mn^+2^ were 0.2 M ammonium chloride, 20% PEG 3350 or 0.1 M succinic acid pH 7.0, 15% PEG3350, respectively. All crystallization experiments took place at 16°C.

### X-ray diffraction data collection, structure determination, and refinement

Crystals were harvested using mounted CryoLoops (Hampton Research, California) and then flash-frozen in liquid nitrogen until data collection. Data for SAH-bound OMT were collected on an in-house FR-E+ Superbright (Rigaku, Washington) rotating anode X-ray generator at a wavelength of 1.54 Å and data for *Apo* OMT was collected at the LS-CAT 21ID-G beam line at the Advanced Photon Source at a wavelength of 0.9786 Å. Data for the SAM-Mn and SAH-Mn complexes were collect at the LS-CAT 21ID-F beam line at the Advanced Photon Source at a wavelength of 0.9787 Å. All data were indexed, integrated, and scaled using the programs XDS and XSCALE [77]. Data statistics for both datasets are available in Table 5. Phases for structure determination of the SAH-bound OMT were obtained via iodide-SAD using the method previously described by Abendroth *et al*. [41]. Heavy atom searches, phasing, and density modification were performed using the programs PHENIX HySS [78], Phaser [79], and SOLVE/RESOLVE [80]. Initial model building into density-modified electron density maps was performed using the program ARP/wARP [81]. Phases for *Apo* OMT were obtained by molecular replacement using the program Phaser, where the SAH-bound OMT structure was used as the search model. Both structures were refined against the reflection data using the programs PHENIX [82] and REFMAC [83] interspersed with rounds of model building using the program Coot [84]. Figs containing molecular graphics were prepared using the program PyMOL (https://www.pymol.org).

### Bioinformatic and phylogenetic analysis of *A*. *phagocytophilum* OMT

To identify the possible origin of the *A*. *phagocytophilum* OMT, the corresponding protein sequence available in GenBank for *A*. *phagocytophilum* isolate HZ (GI:88607321) was used for a PSI-BLAST. The OMTs with the lowest E-values and highest similarity to the *A*. *phagocytophilum* OMT were aligned using ClustalW from MacVector 12.0 (MacVector, Inc, North Carolina). A Minimum Evolution phylogenetic tree of all the OMTs was generated using MEGA 4.0. Conserved motifs within the most closely related non-Anaplasmataceae OMTs, along with the OMT from *A*. *phagocytophilum*, were identified using MEME (http://meme.nbcr.net) [85].

### Putative tertiary structure and localization of methylated residues

Phyre2 [42] was used to determine the putative tertiary structure of the protein (Msp4) that was identified as being methylated by iTRAQ analysis as well as the *in vitro* methylation assay. The putative localization of the modified residues was determined from the protein sequence and the structure generated from Phyre2. Phobius (http://phobius.sbc.su.se/cgi-bin/predict.pl) was used to determine where the modified residues were located within the membrane of the bacteria since Msp4 is a surface protein [61].

### Animals and ethics statement

This study was performed in strict accordance with the recommendations in the Guide for the Care and Use of Laboratory Animals of the National Institutes of Health. None of the procedures used caused more than momentary pain, and infection with *A*. *phagocytophilum* does not cause illness in mice. Animals were euthanized with CO_2_ before collecting blood for production of serum following current AVMA guidelines. The protocol was approved by the institutional Animal Care and Use Committee of the University of Minnesota (Protocol ID: 1303-30435A).

### Accession numbers

The NCBI accession numbers for *A*. *phagocytophilum* proteins mentioned in the body of the article are the following: *O*-methyltransferase (GI:88598384); Major Surface Protein 4 (GI:88607879); Hypothetical protein APH_0406 (GI:88607117); OmpA family member (GI:88607566); P44_18ES (GI:88607256); Major Surface Protein 5 (GI:88607263); DNA-dependent RNA polymerase subunit B (GI:88607105); Antioxidant AhpCTSA family protein (GI:88607183); Ankyrin (GI:88607707); P44-1 outer membrane protein (GI:88607426); OMP85 (GI:88607567); Hypothetical protein APH_0405 (GI:88607654); Chaperonine GrpE (GI:88607566); Chaperonin GroEL (GI:88606723); DnaK (GI:88607549); Cytochrome C oxidase subunit II (GI:88607721); TypA (GI:88607727); Outer membrane protein P44-16b (GI:88607043); Preprotein translocase subunit SecA (GI:886078849).

The NCBI accession numbers for proteins from other organism are the following: *B*. *bacteriovorus* OMT (GI:426402377); *H*. *ochraceum* OMT (GI:262196431); *Anaeromixobacter sp*. OMT (GI:197124171); *C*. M. mitochondrii OMT (GI:339319550); and *Gloeocapsa sp*. (GI:434391334).

The PDB accession numbers for the crystal structures mentioned in the article are the following: *A*. *phagocytophilum* OMT + SAH (4OA5); *A*. *phagocytophilum* OMT *Apo* (4OA8); *A*. *phagocytophilum* OMT + SAM + Mn^+2^ (4PCL); *A*. *phagocytophilum* OMT + SAH + Mn^+2^ (4PCA); and OMT *C*. *synechocystis* (3CBG).

## Supporting Information

S1 Fig Effect of AdOx treatment on *A*. *phagocytophilum* HZ and ΔOMT binding to ISE6 cells. To investigate the possibility that AdOx inhibited methyltransferases involved in infection of ISE6 cells other than the one affected by the mutation, wild-type and ΔOMT bacteria were incubated with different concentrations of the methyltransferase inhibitor AdOx for 1 hr and then binding assays were performed for 1 hr with ISE6 cells. Unbound bacteria were washed away and cells with bound bacteria were examined by immunofluorescence microscopy. A significant decrease in binding similar to that observed previously was only seen in wild-type bacteria (purple bars). Addition of AdOx to ΔOMT (green bars) caused only a small additional decrease in binding, suggesting no other methyltransferases were involved. The bars represent the average number of *A*. *phagocytophilum* HZ wild-type or ΔOMT adherent to ISE6 cells from three replicates. Vertical lines above the bars represent the standard error of the mean. Values for bars labeled with the same letter were not significantly different at p<0.05.
(TIF)

S2 Fig Trypsin protection assay to confirm that ΔOMT enters ISE6 cells Wild-type (ApHzWT) and mutant (ΔOMT) bacteria were allowed to bind to ISE6 cells. After incubation at 34°C for 4 days, infected cells were mechanically detached from the flask by scraping (scraped) or treated with trypsin once (1 x) or twice (2 x) to determine if bacteria were internalized into ISE6 cells. To demonstrate that trypsin removes extracellular bacteria adherent to ISE6 cells, wild-type bacteria were incubated with ISE6 cells for 1 hr to allow binding, and cells then scraped or trypsinized. Bacteria were labeled with FITC (green) and the actin of the cells was labeled with mCherry-LifeAct (red, A-F) or nuclei were labeled with DAPI (blue, G and H) to aid visualization. Arrow heads in panels A, B, and C indicate wild-type morulae; arrows in panels D-H point to individual mutant bacteria; asterisks in panels D, E, and F are used to label host cell nuclei that were recognized by the primary dog-anti *A*. *phagocyophilum* serum. The bar in panel F represents 20 μm, and applies to panels A-F; the bar in panel H represents 10 μm, and applies to panels G and H.
(TIF)

S3 Fig Expression of *A*. *phagocytophilum* HZ wild-type and ΔOMT genes during growth in HL-60 cells compared to wild-type grown in ISE6 cells. Examination of iTRAQ data identified several proteins (Msp4, OmpA, Aph_0404, p44-18ES, Aph_0405, and Cyt C) that were up-regulated in the ΔOMT when incubated with ISE6 cells for 4 hr. Transcription of their encoding genes was examined during infection of HL-60 cells in both *A*. *phagocytophilum* HZ wild-type and ΔOMT, and compared with wild-type transcription during infection of ISE6 cells. Wild-type and ΔOMT bacteria were purified from HL-60 cells and inoculated into either ISE6 cells (wild-type) or HL-60 cell cultures (wild-type and ΔOMT). After 5 days p.i., RNA was purified and qRT-PCR was performed. The values shown are the fold change in expression during late phases of ΔOMT (dark blue bars) and wild-type (light blue bars) bacteria growth in HL-60 cells compared to the gene expression of wild-type *A*. *phagocytophilum* growing in ISE6 cells. The length of the bars represents the average fold expression change from three replicates and the vertical lines represent the standard deviation. The expression of each gene was similar in both the wild-type and the mutant, suggesting that the mutation of the *omt* gene did not affect their transcription during infection of HL-60 cells. Msp4 was down-regulated 0.003-fold, OmpA, Aph_0404, and P44-18ES were up-regulated 19.07-, 4.5-, and 267-fold, respectively, whereas Aph_0405 (1.5-fold change) and Cyt C were unchanged (1.3-fold change). Gene expression was normalized to expression of the single copy gene *msp5*.
(TIF)

S4 Fig Substrate and enzyme concentration dependent increase in the methylation of rMsp4 over time. An in vitro methylation assay using recombinant OMT and recombinant Msp4 was performed to determine the kinetics of the enzyme reaction with increasing concentrations of either the recombinant enzyme or the substrate. A linear decrease in the time to reach Vmax as a function of increased concentrations of A) rOMT or B) rMsp4 was observed in both cases, which was directly proportional to the increase in concentration of the enzyme and the substrate.
(TIF)

S5 Fig Superimposition of *Apo* and SAH-bound structures. The PDB files 4OA5 and 4OA8 had chain A aligned via C_α_ atoms in the program PyMOL (http://www.pymol.org). A stereo view of the aligned files in ribbon format is presented. The *Apo*-bound structure is colored red with yellow for amino acids in positions 31–40, and the SAH-bound structure is colored blue with cyan for amino acids in positions 31–40. SAH from 4OA5 is represented in stick-format to show where ligand binding occurs.
(TIF)

S6 Fig Phylogenetic relationship and motif similarities between homologous OMT enzymes from *A*. *phagocytophilum* and other bacteria. A) Phylogenetic tree showing the relationship between *A*. *phagocytophilum* OMT and it closest homologs present in other members of the *Anaplasmataceae*, *Rickettsiales*, and other bacteria. The tree was generated using protein sequences available in GenBank. Multiple alignments were generated using MacVector 12.0. The relationships were inferred using the minimum evolution criterion, and the distances were computed using the total number of differences. The values shown are from 1000 bootstrap replicates and all positions containing gaps and missing data were eliminated. Members of the *Anaplasmataceae* family are shown in maroon, α-*proteobacteria* are shown in red, Δ-*proteobacteria* are shown in green, *Cyanobacteria* are shown in light blue, *Actinobacteria* are shown in pink, *Bacteriodetes* are shown in dark purple, *Chloroflexi* are shown in medium blue, *Acidobacteria* are shown in dark green, *Firmicutes* are shown in light purple, and γ-proteobacteria are shown in dark blue. According to BLAST results, the *A*. *phagocytophilum* OMT is only found in the *Anaplasmataceae* and *C*. Midichloria mitochondrii, and is absent from all other *Rickettsiales* and α-*proteobacteria* and the most closely OMT is found in the Δ-proteobacterium *Bdellovidrio bacteriovorus*. B) Alignment of motifs present in the amino acid sequences from the 5 non*-Anaplasmataceae* members that are most closely related to the *A*. *phagocytophilum* OMT. An analysis of motifs performed using MEME identified 3 conserved motifs within the OMT protein sequences. The conserved residues in each motif are shown next to the alignments.
(TIFF)

S7 Fig Msp4 putative tertiary structure and position of methylated glutamic acid (E) residues. A) Phyre2 prediction of the putative secondary structure of Msp4 protein sequence as a 14-strand β-barrel porin. The structure was generated by homology with other proteins with known crystal structure. The red square shows the position at the beginning of the 7^th^ β-strand that contains the glutamic acid residues targeted by the OMT. B) Msp4 tertiary structure produced by Phyre2 showing the typical porin-like structure formed by several β-strands. The black arrow points to the position of the glutamic acid residues modified by the OMT. C) Msp4 transmembrane and signal peptide prediction by Phobius. The red line represents the location of a signal peptide predicted for the Msp4 sequence (GI: 88598942), and its probability. The blue line represents the portion of the protein that is non-cytoplasmic and the probability of a correct prediction. Phobius predicted that Msp4 contained a signal peptide within the first 50 amino acids of the protein, which corresponded to the α-helix at the N-terminus predicted by Phyre2.
(TIF)

S8 Fig Reverse-Transcriptase (RT)-PCR of the intergenic regions flanking the *omt* gene. RT-PCR reactions using primers that amplify A) an ~850 bp segment (+ control, arrow head) within the intergenic regions between the *omt* gene and the gene encoding the sensor histidine kinase (APH_0582), and B) a 702 bp segment within the inter-genic region between the *omt* gene and the hypothetical protein encoding gene *APH_0585* to test whether the genes were transcribed as a polycistronic mRNA. Total RNA was extracted from ISE6 cells infected with wild-type *A*. *phagocytophilum* HZ strain at the indicated times. NRT panel: amplification products without reverse-transcriptase. RT panel: amplification products with reverse-transcriptase. Positive control consisted of genomic DNA purified from wild-type bacteria infecting ISE6 cells. Ladder = molecular weight standard. Results do not support co-transcription of *omt* and flanking regions.
(TIFF)

S9 Fig Binding of recombinant Msp4- and OMT-expressing *Escherichia coli* to ISE6 cells. A) To test if methylation of Msp4 was necessary for binding to ISE6 cells, we transformed *E*. *coli* to express either Msp4 alone or the OMT and Msp4. Binding of *E*. *coli* transformed with the empty pET29b vector (negative control), to express Msp4 alone, or both Msp4 and the OMT was measured as the number of bacteria bound per cell. Blue bars represent the average value from three replicates, and the vertical lines indicate the standard deviation. B) Phobius prediction of OMT location within *A*. *phagocytophilum*. Lack of a signal peptide, a greater predicted likelihood of a non-cytoplasmic (blue line) rather than cytoplasmic (green line) location, and absence of transmembrane domains (gray bars) suggested that the OMT might interact with the periplasmic membrane of the bacteria. C) Agarose gel showing RT-PCR amplification of cDNA from *omt* and *msp4* transcripts within transformed *E*. *coli*. Left panel, PCR reaction products using *omt*-specific primers and the following templates: Lanes 1–5, negative controls: lane 1, RNA from *E*. *coli* transformed with the empty expression cassette pET29b; lane 2, RNA from *E*. *coli* expressing Msp4 plus OMT; lane 3, no template; lane 4, cDNA from *E*. *coli* containing empty pET29b; lane 5, cDNA from *E*. *coli* transformed to express Msp4; lane 6, cDNA from *E*. *coli* transformed to express Msp4 and OMT; lane 7 (positive control), DNA from *E*. *coli* transformed to express Msp4 and OMT. Right panel, PCR reaction products using *msp4*-specific primers and the following templates: Lanes 1–5, negative controls: lane 1, RNA from *E*. *coli* transformed with the empty expression cassette pET29b; lane 2, RNA from *E*. *coli* expressing Msp4; lane 3, RNA from *E*. *coli* expressing Msp4 plus OMT; lane 4, no template; lane 5, cDNA from *E*. *coli* containing empty pET29b; lane 6, cDNA from *E*. *coli* transformed to express Msp4; lane 7, cDNA from *E*. *coli* transformed to express Msp4 and OMT; lane 8 (positive control), DNA from *E*. *coli* transformed to express Msp4 and OMT.
(TIF)

S1 Text Protein summary of identified proteins during iTRAQ study of tick cells infected with ΔOMT or wild-type bacteria. (XLSX)

S2 Text Peptide summary of identified peptides during iTRAQ (replicate 1). (TXT)

S3 Text Peptide summary of identified peptides during iTRAQ (replicate 2). (TXT)

S4 Text Peptide summary of identified peptides during iTRAQ (replicate 3). (TXT)

S5 Text Binding assays using *Escherichia coli* transformed with Msp4+OMT, Msp4 alone, or empty vector. (DOC)

S1 Table Protein identity of the subjects with the highest identity and similitude to *A*. *phagocytophilum* retrieved with PSI-BLAST. (DOC)

S2 Table Primers used to determine gene expression using qRT-PCR from *A*. *phagocytophilum* RNA. (DOCX)

S3 Table Primers used for the amplification of *A*. *phagocytophilum* genes for the production of recombinant proteins. (DOCX)

S4 Table *I*. *scapularis* proteins that present a reduction in peptide methylation in the OMT mutant. (DOCX)
