# Supplementary figures and images for: An O-Methyltransferase Is Required for Infection of Tick Cells by Anaplasma phagocytophilum
Source: PLoS Pathog. 2015 Nov 6;11(11):e1005248. doi: 10.1371/journal.ppat.1005248 (PMC4636158; doi:10.1371/journal.ppat.1005248)

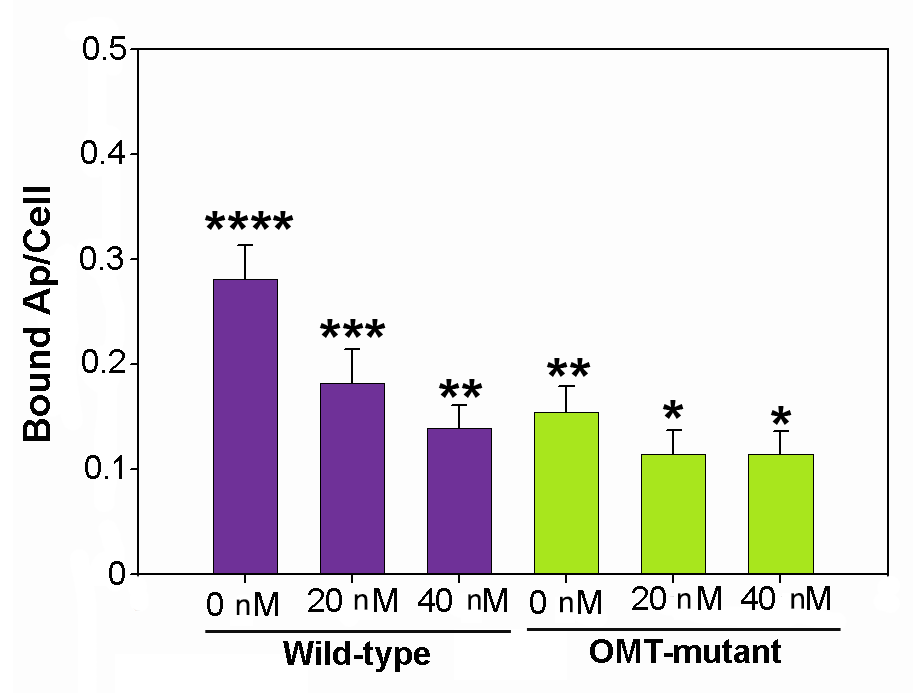

Supplement: S1 Fig — To investigate the possibility that AdOx inhibited methyltransferases involved in infection of ISE6 cells other than the one affected by the mutation, wild-type and ΔOMT bacteria were incubated with different concentrations of the methyltransferase inhibitor AdOx for 1 hr and then binding assays were performed for 1 hr with ISE6 cells. Unbound bacteria were washed away and cells with bound bacteria were examined by immunofluorescence microscopy. A significant decrease in binding similar to that observed previously was only seen in wild-type bacteria (purple bars). Addition of AdOx to ΔOMT (green bars) caused only a small additional decrease in binding, suggesting no other methyltransferases were involved. The bars represent the average number of A. phagocytophilum HZ wild-type or ΔOMT adherent to ISE6 cells from three replicates. Vertical lines above the bars represent the standard error of the mean. Values for bars labeled with the same letter were not significantly different at p<0.05. (TIF) [file ppat.1005248.s001.tif]

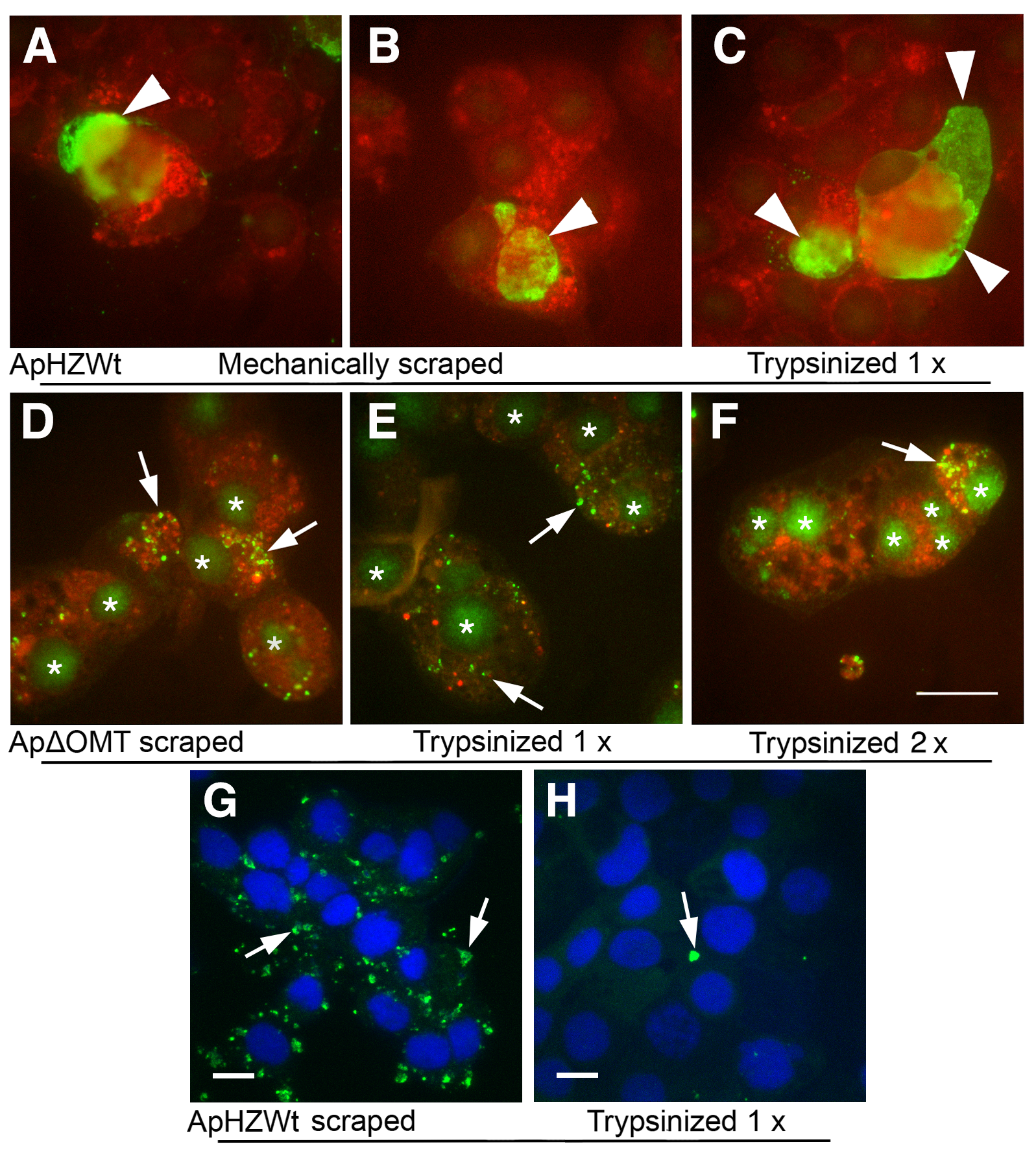

Supplement: S2 Fig — Wild-type (ApHzWT) and mutant (ΔOMT) bacteria were allowed to bind to ISE6 cells. After incubation at 34°C for 4 days, infected cells were mechanically detached from the flask by scraping (scraped) or treated with trypsin once (1 x) or twice (2 x) to determine if bacteria were internalized into ISE6 cells. To demonstrate that trypsin removes extracellular bacteria adherent to ISE6 cells, wild-type bacteria were incubated with ISE6 cells for 1 hr to allow binding, and cells then scraped or trypsinized. Bacteria were labeled with FITC (green) and the actin of the cells was labeled with mCherry-LifeAct (red, A-F) or nuclei were labeled with DAPI (blue, G and H) to aid visualization. Arrow heads in panels A, B, and C indicate wild-type morulae; arrows in panels D-H point to individual mutant bacteria; asterisks in panels D, E, and F are used to label host cell nuclei that were recognized by the primary dog-anti A. phagocyophilum serum. The bar in panel F represents 20 μm, and applies to panels A-F; the bar in panel H represents 10 μm, and applies to panels G and H. (TIF) [file ppat.1005248.s002.tif]

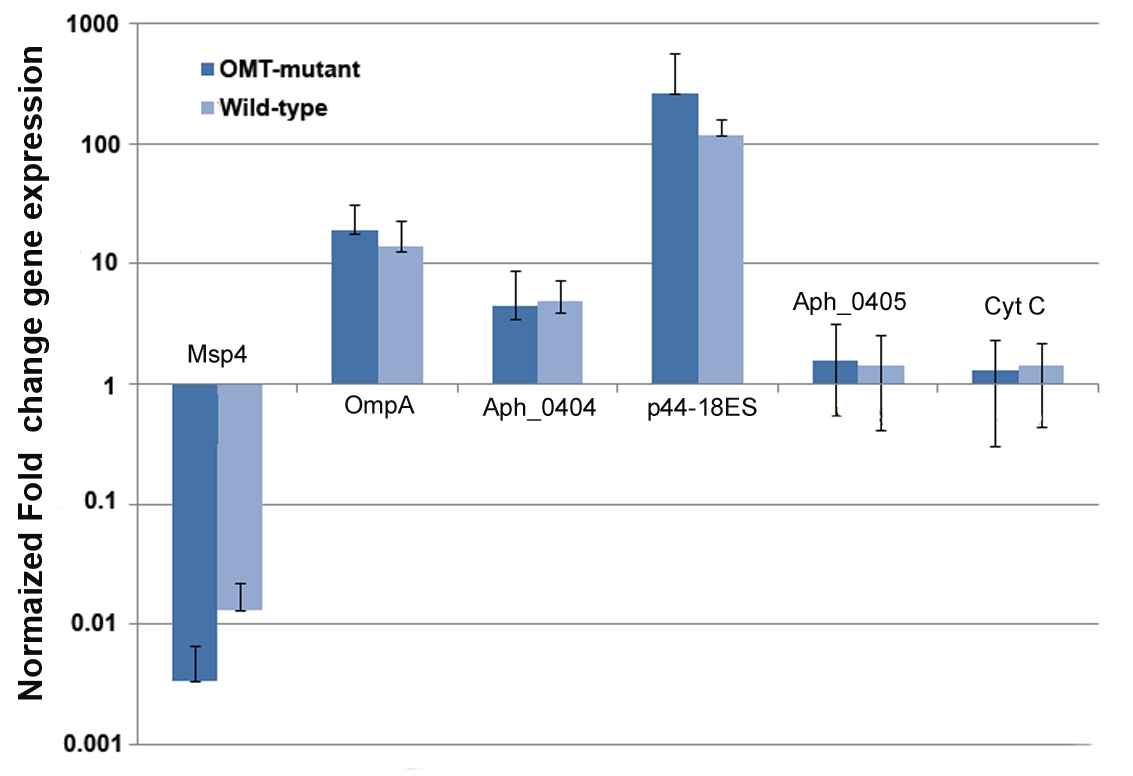

Supplement: S3 Fig — Examination of iTRAQ data identified several proteins (Msp4, OmpA, Aph_0404, p44-18ES, Aph_0405, and Cyt C) that were up-regulated in the ΔOMT when incubated with ISE6 cells for 4 hr. Transcription of their encoding genes was examined during infection of HL-60 cells in both A. phagocytophilum HZ wild-type and ΔOMT, and compared with wild-type transcription during infection of ISE6 cells. Wild-type and ΔOMT bacteria were purified from HL-60 cells and inoculated into either ISE6 cells (wild-type) or HL-60 cell cultures (wild-type and ΔOMT). After 5 days p.i., RNA was purified and qRT-PCR was performed. The values shown are the fold change in expression during late phases of ΔOMT (dark blue bars) and wild-type (light blue bars) bacteria growth in HL-60 cells compared to the gene expression of wild-type A. phagocytophilum growing in ISE6 cells. The length of the bars represents the average fold expression change from three replicates and the vertical lines represent the standard deviation. The expression of each gene was similar in both the wild-type and the mutant, suggesting that the mutation of the omt gene did not affect their transcription during infection of HL-60 cells. Msp4 was down-regulated 0.003-fold, OmpA, Aph_0404, and P44-18ES were up-regulated 19.07-, 4.5-, and 267-fold, respectively, whereas Aph_0405 (1.5-fold change) and Cyt C were unchanged (1.3-fold change). Gene expression was normalized to expression of the single copy gene msp5. (TIF) [file ppat.1005248.s003.tif]

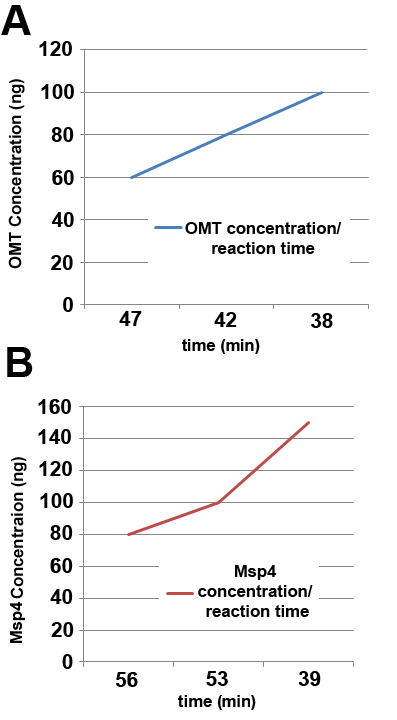

Supplement: S4 Fig — An in vitro methylation assay using recombinant OMT and recombinant Msp4 was performed to determine the kinetics of the enzyme reaction with increasing concentrations of either the recombinant enzyme or the substrate. A linear decrease in the time to reach Vmax as a function of increased concentrations of A) rOMT or B) rMsp4 was observed in both cases, which was directly proportional to the increase in concentration of the enzyme and the substrate. (TIF) [file ppat.1005248.s004.tif]

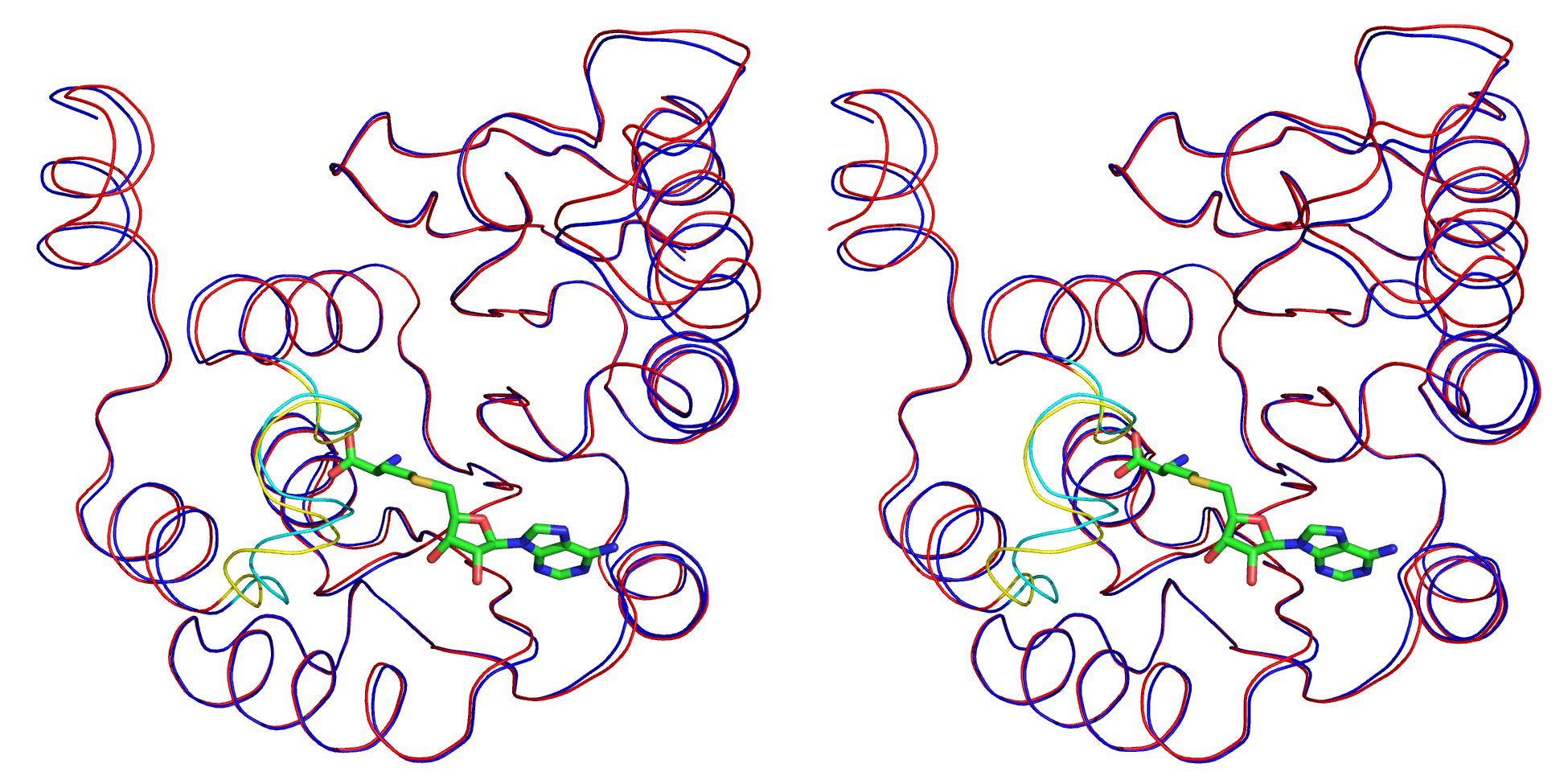

Supplement: S5 Fig — The PDB files 4OA5 and 4OA8 had chain A aligned via Cα atoms in the program PyMOL (http://www.pymol.org). A stereo view of the aligned files in ribbon format is presented. The Apo-bound structure is colored red with yellow for amino acids in positions 31–40, and the SAH-bound structure is colored blue with cyan for amino acids in positions 31–40. SAH from 4OA5 is represented in stick-format to show where ligand binding occurs. (TIF) [file ppat.1005248.s005.tif]

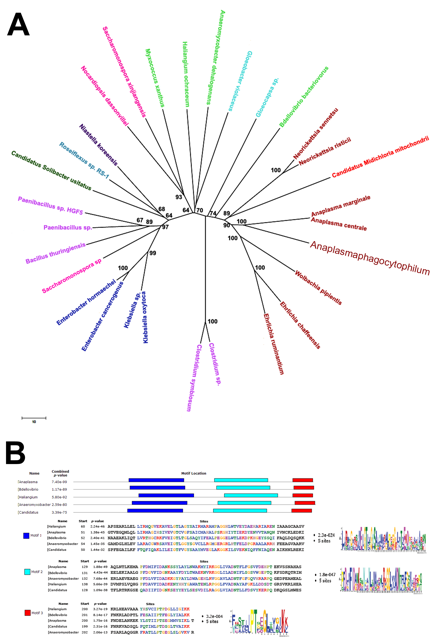

Supplement: S6 Fig — A) Phylogenetic tree showing the relationship between A. phagocytophilum OMT and it closest homologs present in other members of the Anaplasmataceae, Rickettsiales, and other bacteria. The tree was generated using protein sequences available in GenBank. Multiple alignments were generated using MacVector 12.0. The relationships were inferred using the minimum evolution criterion, and the distances were computed using the total number of differences. The values shown are from 1000 bootstrap replicates and all positions containing gaps and missing data were eliminated. Members of the Anaplasmataceae family are shown in maroon, α-proteobacteria are shown in red, Δ-proteobacteria are shown in green, Cyanobacteria are shown in light blue, Actinobacteria are shown in pink, Bacteriodetes are shown in dark purple, Chloroflexi are shown in medium blue, Acidobacteria are shown in dark green, Firmicutes are shown in light purple, and γ-proteobacteria are shown in dark blue. According to BLAST results, the A. phagocytophilum OMT is only found in the Anaplasmataceae and C. Midichloria mitochondrii, and is absent from all other Rickettsiales and α-proteobacteria and the most closely OMT is found in the Δ-proteobacterium Bdellovidrio bacteriovorus. B) Alignment of motifs present in the amino acid sequences from the 5 non-Anaplasmataceae members that are most closely related to the A. phagocytophilum OMT. An analysis of motifs performed using MEME identified 3 conserved motifs within the OMT protein sequences. The conserved residues in each motif are shown next to the alignments. (TIFF) [file ppat.1005248.s006.tiff]

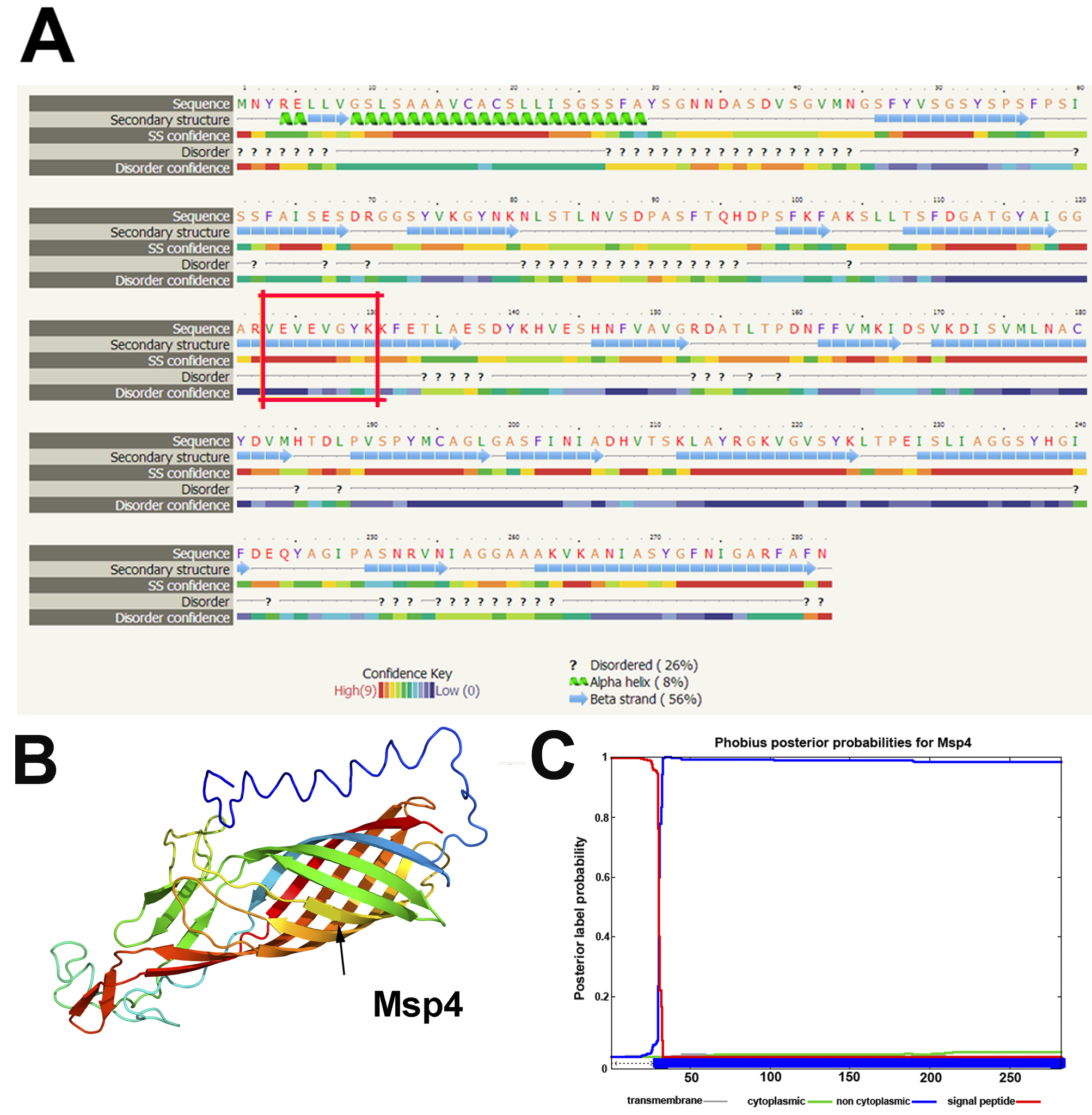

Supplement: S7 Fig — A) Phyre2 prediction of the putative secondary structure of Msp4 protein sequence as a 14-strand β-barrel porin. The structure was generated by homology with other proteins with known crystal structure. The red square shows the position at the beginning of the 7th β-strand that contains the glutamic acid residues targeted by the OMT. B) Msp4 tertiary structure produced by Phyre2 showing the typical porin-like structure formed by several β-strands. The black arrow points to the position of the glutamic acid residues modified by the OMT. C) Msp4 transmembrane and signal peptide prediction by Phobius. The red line represents the location of a signal peptide predicted for the Msp4 sequence (GI: 88598942), and its probability. The blue line represents the portion of the protein that is non-cytoplasmic and the probability of a correct prediction. Phobius predicted that Msp4 contained a signal peptide within the first 50 amino acids of the protein, which corresponded to the α-helix at the N-terminus predicted by Phyre2. (TIF) [file ppat.1005248.s007.tif]

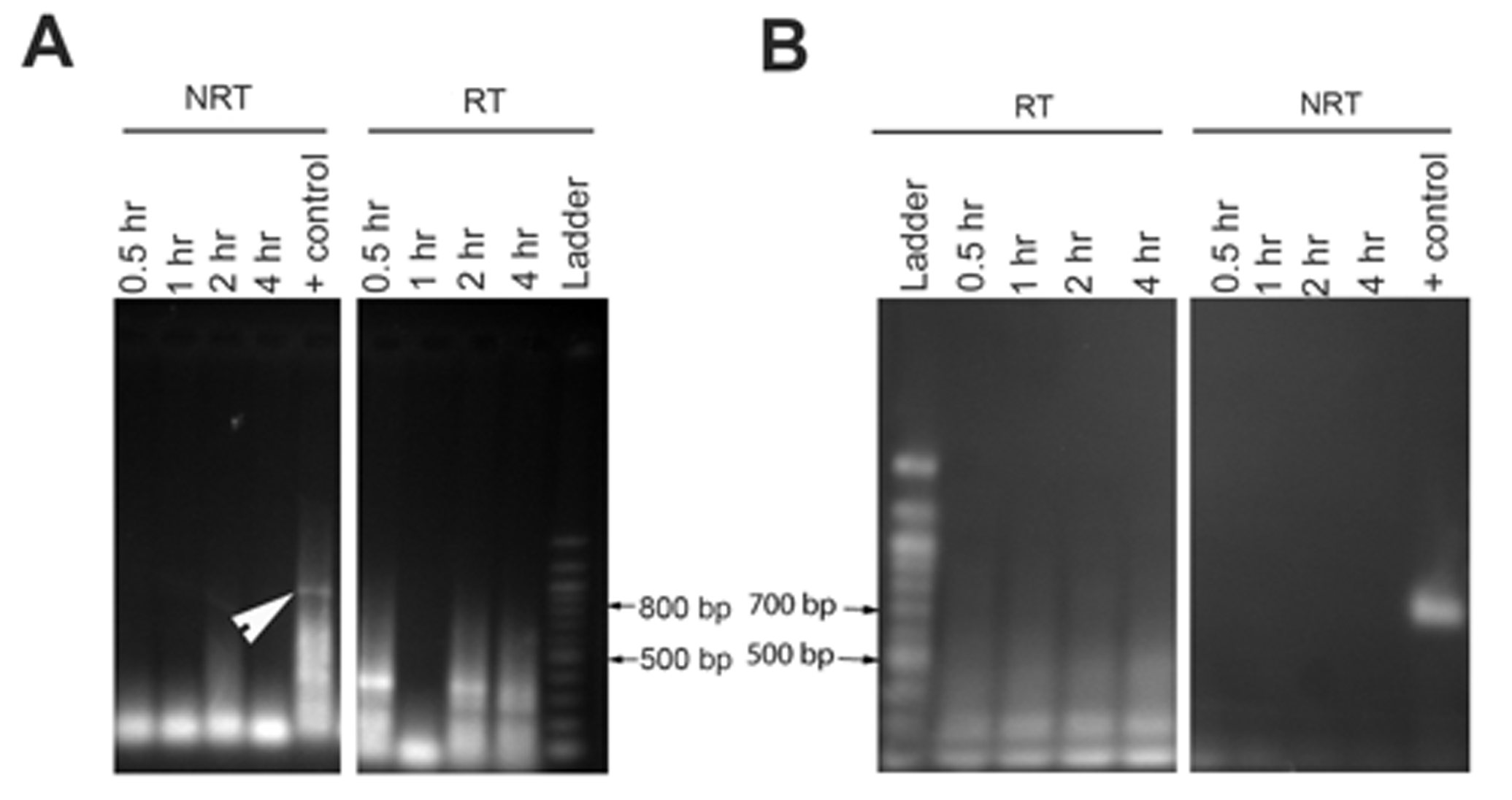

Supplement: S8 Fig — RT-PCR reactions using primers that amplify A) an ~850 bp segment (+ control, arrow head) within the intergenic regions between the omt gene and the gene encoding the sensor histidine kinase (APH_0582), and B) a 702 bp segment within the inter-genic region between the omt gene and the hypothetical protein encoding gene APH_0585 to test whether the genes were transcribed as a polycistronic mRNA. Total RNA was extracted from ISE6 cells infected with wild-type A. phagocytophilum HZ strain at the indicated times. NRT panel: amplification products without reverse-transcriptase. RT panel: amplification products with reverse-transcriptase. Positive control consisted of genomic DNA purified from wild-type bacteria infecting ISE6 cells. Ladder = molecular weight standard. Results do not support co-transcription of omt and flanking regions. (TIFF) [file ppat.1005248.s008.tiff]

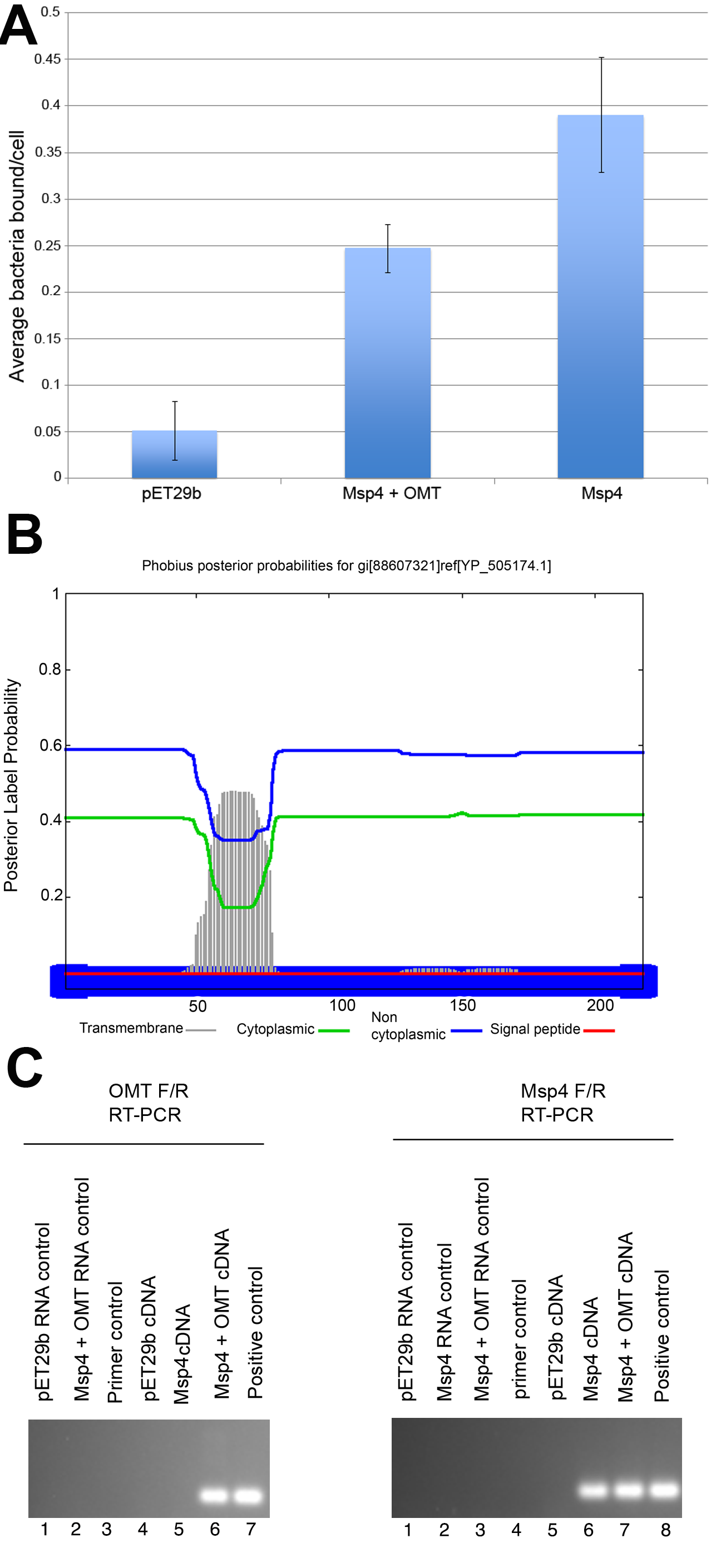

Supplement: S9 Fig — A) To test if methylation of Msp4 was necessary for binding to ISE6 cells, we transformed E. coli to express either Msp4 alone or the OMT and Msp4. Binding of E. coli transformed with the empty pET29b vector (negative control), to express Msp4 alone, or both Msp4 and the OMT was measured as the number of bacteria bound per cell. Blue bars represent the average value from three replicates, and the vertical lines indicate the standard deviation. B) Phobius prediction of OMT location within A. phagocytophilum. Lack of a signal peptide, a greater predicted likelihood of a non-cytoplasmic (blue line) rather than cytoplasmic (green line) location, and absence of transmembrane domains (gray bars) suggested that the OMT might interact with the periplasmic membrane of the bacteria. C) Agarose gel showing RT-PCR amplification of cDNA from omt and msp4 transcripts within transformed E. coli. Left panel, PCR reaction products using omt-specific primers and the following templates: Lanes 1–5, negative controls: lane 1, RNA from E. coli transformed with the empty expression cassette pET29b; lane 2, RNA from E. coli expressing Msp4 plus OMT; lane 3, no template; lane 4, cDNA from E. coli containing empty pET29b; lane 5, cDNA from E. coli transformed to express Msp4; lane 6, cDNA from E. coli transformed to express Msp4 and OMT; lane 7 (positive control), DNA from E. coli transformed to express Msp4 and OMT. Right panel, PCR reaction products using msp4-specific primers and the following templates: Lanes 1–5, negative controls: lane 1, RNA from E. coli transformed with the empty expression cassette pET29b; lane 2, RNA from E. coli expressing Msp4; lane 3, RNA from E. coli expressing Msp4 plus OMT; lane 4, no template; lane 5, cDNA from E. coli containing empty pET29b; lane 6, cDNA from E. coli transformed to express Msp4; lane 7, cDNA from E. coli transformed to express Msp4 and OMT; lane 8 (positive control), DNA from E. coli transformed to express Msp4 and OMT. (TIF) [file ppat.1005248.s009.tif]
