# Supplementary material for: An O-Methyltransferase Is Required for Infection of Tick Cells by Anaplasma phagocytophilum
Source: PLoS Pathog. 2015 Nov 6;11(11):e1005248. doi: 10.1371/journal.ppat.1005248 (PMC4636158; doi:10.1371/journal.ppat.1005248)
Supplement: S5 Text — (DOC) [file ppat.1005248.s014.doc]

**Supplemental Material and Methods**

**Binding of *Escherichia coli* expressing recombinant Msp4 to ISE6 cells**

The entire Msp4 sequence was cloned into pEMB85 and pEMB86 vectors replacing the signal peptide of Msp4 with the signal peptide from OmpA of *E. coli.* Additionally, four different constructs were designed with 4 different combinations of mutations within the C-terminus of Msp4 to facilitate localization to the outer membrane of the *E. coli.* The *omt* CDS was cloned into pET28b behind the T7 promoter. The *omt* CDS with the T7 promoter was PCR amplified, sequence verified, and transferred to the Msp4 expression plasmids.

*E. coli* (BL21 [DE3]) containing either of two expression constructs, one with *omt* and *msp4* (10975 + OMT) and the other with *msp4* alone (10975), were tested for their ability to bind to ISE6 cells compared to control bacteria containing the expression cassette without either of the genes, as follows. Three colonies containing each of the two test constructs and three control colonies (expression construct without *msp4* or *omt*) were picked, cultured at 34°C in 2X YT broth for 2 hours (OD: 0.135 – 0.256) , then the cultures were induced by adding 100 µM IPTG and incubating for an additional two hours.

Bacterial concentrations of the nine *E. coli* cultures were equalized by diluting with culture medium until each had an OD of 0.5 at 600 nm. Ten microliters (4x10^6 bacteria) of each was added to 100 µl ISE6 growth medium containing 2x10^5 suspended ISE6 cells (20 bacteria/cell) and incubated for 1 hr at 34°C. Cells were washed twice, then a cytospin slide of each replicate was made and giemsa stained. Bacteria binding per cell was determined by counting the number of bacteria on at least 100 cells in each replicate.

**Verification of *omt* and *msp4* expression in *E. coli***

pET29b, 10975 and 10975 + OMT in BL21 (DE3) cells (NEB) were each inoculated into 3 ml 2XYT media containing 50µg/ml kanamycin and incubated for 4 hours at 34°C, then induced with 100µM IPTG and incubated for an additional 2 hours. A 1 ml sample was collected and pelleted 2 hours post- induction. The pellets were re-suspended in 1 ml TRI Reagent® (Sigma) and RNA was purified using the Direct-zol RNA MiniPrep kit (Zymo Research) as per manufacturer’s protocol. RNA samples were DNased 5 times using Turbo DNA-free kit (Life Technologies), and purified between DNase treatments with the RNA Clean and Concentrator kit (Zymo Research) using the manufacturer’s rigorous DNase treatment protocol due to high DNA contamination. The inactivation reagent was not used as it causes reduced efficiency of DNase with repeated treatments.

SuperScript® II Reverse Transcriptase (Invitrogen) was used for first strand synthesis using 528 ng RNA as template for each sample, using gene specific primers. Each RNA sample was primed using the following reverse strand primers: OMT R (5’-TGC GGA AAA TCC CAC ACA AG-3’), OMT R2 (5’-GCC TTC GCT AGT AGG TAT AAT GG-3’), MSP4 R (5’-GAG ATA GAC GGG AAA GAC GGA GAG-3’), and MSP4 R2 (5’-CCG ATG TTG AAG CCG TAA GA-3’). The resulting cDNA (52.8 ng) was PCR-amplified with GoTaq® DNA polymerase (Promega) according to manufacturer’s protocol. RNA samples (52.8 ng) were also used as template with each primer set to check for DNA contamination. The following forward primers were used in conjunction with the above listed reverse primers at 150nM final concentration: OMT F (5’-GCA CCT TCT GAG TTG CGT ATG G-3’), OMT F2 (5’-TCC TCT TTG GCA GCG TAT TT –‘3), MSP4 F (5’-CAT CAT CAT CAT CAC AGC AGC G-3’), and MSP4 F2 (5’- CTT CTA CCA CGG CAT CTT TGA-3’). Cycling conditions were 1 cycle at 95°C for 2 min; 40 cycles at 95°C for 30 sec, 54°C for 30 sec and 72°C for 1 min, and 1 final cycle at 72°C for 10 min. Amplicons were electrophoresed on 2% agarose and post-stained with GelGreen (Biotium, Hayward, CA).

**Supplemental Results
*E. coli* expressing *msp4* and *omt*, ISE6 binding**

The expression of the *omt* and *msp4* was confirmed by RT-PCR. *msp4* was expressed by both 10975 and 10975 + OMT, whereas the *omt* was expressed only by 10975 + OMT (Figure S10C). Amplicons were not detected in any of the controls. The binding ability of *E. coli* expressing Msp4 and the OMT was evaluated and compared to bacteria expressing Msp4 alone or transformed only with empty pET29b. The three different *E. coli* transformants were each incubated in triplicate with ISE6 cells for 1 hr at 34°C and counted. *E. coli* expressing Msp4 alone bound more to ISE6 with an average of 0.44 bacteria bound/cell whereas an average of 0.24 bacteria bound/cell were observed in the transformants bearing both Msp4 and the OMT (Figure S10A). The control *E. coli* containing pET29b alone bound poorly to ISE6 with only an average of 0.08 bacteria bound/cell observed (Figure S10A).

To determine the possible causes for the lower binding of the *E. coli* co-expressing Msp4 and OMT, we analyzed the OMT in Phobius to establish the possible localization of the enzyme and the possible site where Msp4 methylation takes place in *Anaplasma phagocytophilum.* Phobius determined that the OMT is non-cytoplamic and methylation of Msp4 probably occurs in the periplasm (Figure S10B). Thus, it is probable that the OMT cannot reach the periplasm in *E. coli,* so Msp4 was not methylated. Additionally, the bacteria that are co-expressing Msp4 and OMT are using the same type of promoter (T7) for both genes, and may produce less Msp4 than the *E. coli* expressing only Msp4. This may explain the reduced binding when compared to the *E. coli* expressing only Msp4.
