# Supplementary material for: An O-Methyltransferase Is Required for Infection of Tick Cells by Anaplasma phagocytophilum
Source: PLoS Pathog. 2015 Nov 6;11(11):e1005248. doi: 10.1371/journal.ppat.1005248 (PMC4636158; doi:10.1371/journal.ppat.1005248)
Supplement: S1 Table — (DOC) [file ppat.1005248.s015.doc]

**Table S1. Protein identity of the subjects with the highest identity and similitude to *A. phagocytophilum* retrieved with PSI-BLAST**

| **Organism** | **Protein** | **Protein ID** | **E-value** | **Score (bits)** |
| --- | --- | --- | --- | --- |
| Anaplasma marginale | hypothetical protein AMF_370 | GI:222475076 | 4E-80 | 249 |
| Anaplasma centrale | O-methyltransferase | GI:269958872 | 2E-79 | 248 |
| Ehrlichia ruminantium | O-methyltransferase | GI:58617067 | 3E-68 | 219 |
| Ehrlichia chaffeensis | O-methyltransferase | GI:88657893 | 4E-68 | 219 |
| Wolbachia endosymbiont of Culex quinquefasciatus | O-methyltransferase family protein | GI:190571337 | 2E-63 | 207 |
| Wolbachia endosymbiont of Drosophila melanogaster | O-methyltransferase family protein | GI:42520865 | 5E-61 | 200 |
| Wolbachia endosymbiont of Onchocerca volvulus | O-methyltransferase | GI:111035805 | 5E-58 | 192 |
| Neorickettsia sennetsu | O-methyltransferase family protein | GI:88608149 | 4E-42 | 152 |
| Neorickettsia risticii | O-methyltransferase family protein | GI:254796950 | 3E-41 | 148 |
| Bdellovibrio bacteriovorus | O-methyltransferase | GI:426402377 | 4E-40 | 146 |
| Haliangium ochraceum | O-methyltransferase family protein | GI:262196431 | 1E-39 | 143 |
| Anaeromyxobacter sp. K | O-methyltransferase family protein | GI:197124171 | 1E-36 | 138 |
| Candidatus Midichloria mitochondrii IricVA | O-methyltransferase family protein | GI:339319550 | 2E-36 | 137 |
| Gloeocapsa sp. PCC 7428 | O-methyltransferase family 3 | GI:434391334 | 2E-32 | 126 |
| Klebsiella oxytoca | hypothetical protein HMPREF9686_01422 | GI:423102816 | 7E-32 | 125 |
| Gloeobacter violaceus PCC 7421 | O-methyltransferase | GI:37522385 | 3E-31 | 124 |
| Roseiflexus sp. RS-1 | O-methyltransferase family protein | GI:148657074 | 3E-31 | 123 |
| Enterobacter cancerogenus | O-methyltransferase | GI:261339745 | 2E-30 | 121 |
| Enterobacter hormaechei | O-methyltransferase | GI:334124011 | 1E-29 | 119 |
| Saccharomonospora xinjiangensis | putative O-methyltransferase | GI:383828057 | 3E-29 | 118 |
| Nocardiopsis dassonvillei | O-methyltransferase family 3 | GI:297559607 | 4E-29 | 118 |
| Bacillus thuringiensis | O-methyltransferase | GI:75758590 | 6E-29 | 117 |
| Clostridium sp. | O-methyltransferase family protein | GI:283796163 | 9E-29 | 117 |
| Niastella koreensis | O-methyltransferase family protein | GI:375143786 | 1E-28 | 117 |
| Coniophora puteana | O-methyltransferase family 3 protein | GI:392596339 | 3E-28 | 115 |
