# Supplementary material for: An O-Methyltransferase Is Required for Infection of Tick Cells by Anaplasma phagocytophilum
Source: PLoS Pathog. 2015 Nov 6;11(11):e1005248. doi: 10.1371/journal.ppat.1005248 (PMC4636158; doi:10.1371/journal.ppat.1005248)
Supplement: S2 Table — (DOCX) [file ppat.1005248.s016.docx]

| **Primer name** | **Primer sequence** | **Target** | **Product size** | **Type** |
| --- | --- | --- | --- | --- |
| O-methyltrasf APH_0584 F1 | 5'- CAGTTGGGTGCTCATCAAATAC -3' | *omt* | 121 bp | Unknown |
| O-methyltrasf APH_0584 R1 | 5'- GCAAACAAATCCTCATACCTTG -3' |  |  |  |
| p44-18ES F2 | 5'- GGTGTGTGAGACAAAGCGG -3' | *p44-18ES* | 216 bp | Unknown |
| p44-18ES R2 | 5'- CTCGACGTAGGCCAGTTCT -3' |  |  |  |
| aph0404 F2 | 5'- ATTCTGCGCTGCTGTACCC -3' | *aph_0404* | 210 bp | Unknown |
| aph0404 R2 | 5'- ACAAATCGTCCGCATACC -3' |  |  |  |
| Msp4 F2 | 5'- CGTCTGATGTTAGCGGTG -3' | *msp4* | 205 bp | Unknown |
| Msp4 R2 | 5'- TTAGCGAACTTGAATGAGG -3' |  |  |  |
| OmpA F1 | 5'- GGGACCCTTTAGATCGTACT -3' | *ompA* | 120 bp | Unknown |
| OmpA R1 | 5'- CTTCCAGATAGTAACGTCGG -3' |  |  |  |
| CytC F1 | 5'- TCAGTGGTACTGGTCCTATG -3' | *cytC* | 187 bp | Unknown |
| CytC R1 | 5'- CACAGCCCAGCTATGTATAA - 3' |  |  |  |
| aph0405 F2 | 5'- GAAGGCTAGGGATATAGTGC -3' | *aph_0405* | 273 bp | Unknown |
| aph0405 R2 | 5'- GTAGCAGAGAAAGAACCAATCG -3' |  |  |  |
| rpoB APH_1024 F1 | 5'- CTTTATCCTGCTTTAGAACAACATC -3' | *rpoB* | 286 bp | Normalizer |
| rpoB APH_1024 R1 | 5'- GGTCCGTATGGTCTGGTTACT -3' |  |  |  |
| msp5 F1 | 5'- TGACACTGTGGTTGAACAAGC -3' | *msp5* | 126 bp | Normalizer |
| msp5 R1 | 5'- GAAGAAAAGCCGAACATAAGC -3' |  |  |  |
| 23s F | 5'- GCGCTAAGAGGTGATGAAGG -3' | *23s rRNA* | 267 bp | Normalizer |
| 23s R | 5'- TTCCAAGGCATTCCAGTTTC -3' |  |  |  |

**Table S2. Primers used to determine gene expression using qRT-PCR from *A. phagocytophilum* RNA.**
