# Supplementary material for: An O-Methyltransferase Is Required for Infection of Tick Cells by Anaplasma phagocytophilum
Source: PLoS Pathog. 2015 Nov 6;11(11):e1005248. doi: 10.1371/journal.ppat.1005248 (PMC4636158; doi:10.1371/journal.ppat.1005248)
Supplement: S3 Table — (DOCX) [file ppat.1005248.s017.docx]

| **­­­­­Primer name** | **Primer sequence** | **Protein target** | **Annealing Temp** |
| --- | --- | --- | --- |
| rOMT Fw | 5' GCT AGA GTC GAC TCA TGT GAG CTT TA '3 | GI:88598384 | 40* and 47°C** |
| rOMT Rv | 5' GCT CAG ATA TCG TGC GCA ATG TCT CT '3 | GI:88598384 | 40* and 47°C** |
| rOMTns Fw | 5' GCC AGA TCA TAT GGT GCG CAA TGT CT '3 | GI:88598384 | 45* and 54 °C** |
| rOMTns Rv | 5' CTT TAG CGA ATT CCA TGT GAG CTT TAT '3 | GI:88598384 | 45* and 54 °C** |
| TypA Fw | 5' TGA TCC TCG AGT GCA GCA TAT CCA TGG AAC AAT C '3 | GI:88607727 | 43* and 53°C** |
| TypA Rv | 5' GGA TCC ATA TGA TGT CGA GTG CCT ACG ATT CCA '3 | GI:88607727 | 43* and 53°C** |
| p4416b Fw | 5' ATA GCT CGA GCC TAA CAC CAA ATT CCC CAC CGA C '3 | GI:88607043 | 50* and 60°C** |
| p4416b Rv | 5' CAT ATG GTC ATG GCT GGG ACT GAT GTC AGG GCT '3 | GI:88607043 | 50* and 60°C** |
| msp4 Fw | 5' GTA GTG CAT ATG TAC AGA GAA TTG CTG GT '3 | GI:88607879 | 45* and 52°C** |
| msp4 Rv | 5' CGT AGC CTC GAG TCT TGC TCC TAT GTT GAA GCC G '3 | GI:88607879 | 45* and 52°C** |
| aph0406 Fw | 5' GAT AGC ATA TGA TAG TAG CGG TAA AAC GAG TA '3 | GI:88607117 | 43* and 55°C** |
| aph0406 Rv | 5' GTA TGT CTC GAG TAT AAC TTG ACC TCT ATT TAC AC '3 | GI:88607117 | 43* and 55°C** |
| Aph0906 1^st^ Fw | 5’- ATG GTA GCA TAT GAT GAC TCT GCT GCT TAA GCC AAA C – 3’ | GI:88606911 | 46* and 56°C** |
| Aph0906 1^st^ Rv | 5’ - GTG ATG CCT CGA GAT CGA TCA GAG TGT CAC CGA GCA T – 3’ | GI:88606911 | 46* and 56°C** |

**Table S3. Primers used for the amplification of *A. phagocytophilum* genes for the production of recombinant proteins.**

* Annealing temperature for first 10 cycles. ** Annealing temperature for following 20 cycles.
