# Supplementary material for: An O-Methyltransferase Is Required for Infection of Tick Cells by Anaplasma phagocytophilum
Source: PLoS Pathog. 2015 Nov 6;11(11):e1005248. doi: 10.1371/journal.ppat.1005248 (PMC4636158; doi:10.1371/journal.ppat.1005248)
Supplement: S4 Table — (DOCX) [file ppat.1005248.s018.docx]

| **Accession Number** | **Protein ID** | **Peptides with modifications** | **Ratio Wt:KO** |
| --- | --- | --- | --- |
| **GI241044082** | 3-phosphoglycerate kinase, putative | ALDNPSRPFLAILGGAK Dimethyl(R)@7 | 0.4780 |
| **GI241157545*** | Actin, putative | YPIEHGIVTNWDDMEK Methyl(H)@6 | 0.7378 |
| **GI241326700** | Cell division protein, putative | LYEFPCDDEEENKR Methyl(D)@8 | 0.6880 |
| **GI241104748** | Fasciclin | SFFNNMLLQTAEGDDKIR Methyl(D)@14 | 0.6323 |
| **GI241703753** | Flavonol reductase/cinnamoyl-CoA reductase, putative | EVLEIEPR Methyl(E)@6 Methyl(E)@4 LLLEDGQLR Methyl(E)@4 | 0.6495 0.6523 |
| **GI241558809** | Glycoprotein gC1qBP, putative | IEGFDVK Methyl(D)@5 | 0.6467 |
| **GI241263225** | Heatshock protein 20.6, putative | DGILTIEAPLPALEAPNR Methyl(E)@14 | 0.2999 |
| **GI241830514*** | Hsp90 protein, putative | DQVANSAFVER Methyl(D)@1 LMKDILDIL Methyl(D)@7 | 0.7276 0.5119 |
| **GI241600204** | Phosphatidylinositol-4-phosphate 5-kinase type II, putative | AEQEAVER Methyl(E)@7 | 0.5439 |
| **GI241586870** | Plexin domain-containing protein, putative | DLPVPVTEIPDK Methyl(D)@1 | 0.6624 |
| **GI240974259** | Prolyl 4-hydroxylase alpha subunit, putative | GDDGDVPMDEAAVGK Methyl(E)@10 | 0.6978 |
| **GI241627626** | RNA-Binding protein musashi | RGGGGGASGGGGGYHPYSR Dimethyl(R)@1 | 0.4190 |
| **GI241720809*** | TraB domain-containing protein, putative | AVQEAEK Methyl(E)@4 | 0.4417 |
| **GI242004214** | Ubiquitin-activating enzyme E1, putative | ITAHENR Methyl(N)@6 | 0.6694 |
| **GI241061134** | Voltage-dependent anion-selective channel, putative | VNASLETK Methyl(E)@6 | 0.6737 |

**Table S4.** ***I. scapularis* proteins that present a reduction in peptide methylation in the OMT mutant**
